# Ch^O^P-CT: quantitative morphometrical analysis of the Hindbrain Choroid Plexus by X-ray micro-computed tomography

**DOI:** 10.1186/s12987-023-00502-8

**Published:** 2024-01-24

**Authors:** Viktória Parobková, Petra Kompaníková, Jakub Lázňovský, Michaela Kavková, Marek Hampl, Marcela Buchtová, Tomáš Zikmund, Jozef Kaiser, Vítězslav Bryja

**Affiliations:** 1 Central European Institute of Technology, Brno University of Technology, Brno, Czech Republic; 2 Department of Experimental Biology, Faculty of Science, Masaryk University, 625 00 Brno, Czech Republic; 3 Laboratory of Molecular Morphogenesis, Institute of Animal Physiology and Genetics, Czech Academy of Sciences, 602 00 Brno, Czech Republic; 4 Department of Cytokinetics, Institute of Biophysics, Academy of Sciences of the Czech Republic, Brno, Czech Republic

**Keywords:** Hindbrain choroid plexus, X-ray micro-computed tomography, 3D visualization, Morphometrics

## Abstract

The Hindbrain Choroid Plexus is a complex, cerebrospinal fluid-secreting tissue that projects into the 4th vertebrate brain ventricle. Despite its irreplaceability in the development and homeostasis of the entire central nervous system, the research of Hindbrain Choroid Plexus and other Choroid Plexuses has been neglected by neuroscientists for decades. One of the obstacles is the lack of tools that describe the complex shape of the Hindbrain Choroid Plexus in the context of brain ventricles. Here we introduce an effective tool, termed Ch^O^P-CT, for the noninvasive, X-ray micro-computed tomography-based, three-dimensional visualization and subsequent quantitative spatial morphological analysis of developing mouse Hindbrain Choroid Plexus. Ch^O^P-CT can reliably quantify Hindbrain Choroid Plexus volume, surface area, length, outgrowth angle, the proportion of the ventricular space occupied, asymmetries and general shape alterations in mouse embryos from embryonic day 13.5 onwards. We provide evidence that Ch^O^P-CT is suitable for the unbiased evaluation and detection of the Hindbrain Choroid Plexus alterations within various mutant embryos. We believe, that thanks to its versatility, quantitative nature and the possibility of automation, Ch^O^P-CT will facilitate the analysis of the Hindbrain Choroid Plexus in the mouse models. This will ultimately accelerate the screening of the candidate genes and mechanisms involved in the onset of various Hindbrain Choroid Plexus-related diseases.

**Supplementary Information:**

The online version contains supplementary material available at 10.1186/s12987-023-00502-8.

## Background

The Hindbrain Choroid Plexus is a fragile tissue localized in the vertebrate central nervous system where it occupies the 4th brain ventricle. Together with other Choroid Plexuses placed in the remaining brain cavities, the Hindbrain Choroid Plexus serves a myriad number of roles during embryogenesis and adulthood. Besides the CNS monitoring ensured by the highly specialized immune cells [1, 2], the Hindbrain Choroid Plexus tissue participates in the production and modulation of the cerebrospinal fluid composition [3]. This clear body liquid is essential not only for hydromechanical support of the central nervous system but also for its nourishment and waste removal character [4]. Intriguingly, the cerebrospinal fluid flow ensures the supply of Choroid Plexus-derived morphogens to the adjacent neuronal tissue where it triggers the proliferation and differentiation of the cerebellum progenitors [5, 6].

Such a spectrum of the Hindbrain Choroid Plexus functions is possible only thanks to the unique Hindbrain Choroid Plexus spatial position and highly sophisticated three-dimensional (3D) shape projecting noticeably into the lumen of the 4th ventricle. In fact, the Hindbrain Choroid Plexus represents the most complex and simultaneously the most evolutionary conserved Choroid Plexus shape compared to other Choroid Plexuses [7]. Additionally, the Hindbrain Choroid Plexus tissue is the first Choroid Plexus to occur in the brain ventricular space as it starts to bud early during central nervous system formation, in mice at embryonic day (E) ~ 11.5 [8, 9]. This is also the developmental time point around which the primary Hindbrain Choroid Plexus3D structure, formed by defined numbers of Hindbrain Choroid Plexus branches, is gradually determined. Later, the lateral segments of the Hindbrain Choroid Plexus branches progressively extend into the lateral recesses of the 4th ventricle, which leads to the establishment of the sufficient secretory surface area essential for the proper Hindbrain Choroid Plexus functioning [6, 9].

Considering the Hindbrain Choroid Plexus's delicate character, highly convoluted shape and embedded position deeply in the central nervous system, Hindbrain Choroid Plexus 3D visualization and morphological characterization are not trivial tasks. Not surprisingly the first decent Hindbrain Choroid Plexus or general Choroid Plexus reconstructions have started to appear just recently [10–15]. Nonetheless, these studies are usually burdened by either the low resolution [12, 13] or long sample preparation and tedious manual segmentation [10]. On top of this, these studies usually do not fully exploit the whole information potential of the Choroid Plexus 3D model, as they focus solely on volume analysis and linking volume alternation with various neurodegenerative diseases [14, 15].

Further Choroid Plexus morphological assessment has been done merely on the histological slides, where authors standardized several morphological parameters (total Choroid Plexus area, number and the length of the Hindbrain Choroid Plexus branches [6, 9] or the length of the whole Choroid Plexus epithelium [16]) serving for the detection of the impaired Choroid Plexus growth and morphological maturation within the various mutant mouse models. However, these studies are usually performed just on a few consecutive sections of the Choroid Plexus, thus the complex or regionalized 3D details [2], as well as spatial Choroid Plexus information, are lost.

Spatial Hindbrain Choroid Plexus data like outgrowth angle [17, 18] or the Choroid Plexus placement within the 4th ventricle [6, 9] are gradually incorporated into the prognosis of the various Choroid Plexus -related diseases (e.g. Vermian hypoplasia [17] or Blake's pouch cyst [18]) or served as a crucial part in the disclosure of the full Choroid Plexus potential and its sphere of influence within the central nervous system (e.g. Choroid Plexus -mediated morphogenesis of the cerebellum [5, 6]). Thus, all shortcomings and gaps of the above-mentioned traditional histological evaluation or more advanced 3D reconstructions of the Choroid Plexus must be solved promptly to enhance our knowledge about the Hindbrain Choroid Plexus tissue and its involvement in the onset and progress of neurological disorders.

Here, we present a novel sophisticated tool—Ch^O^P-CT (Ch^O^Pping the Ch^O^roid Plexus out of the X-ray micro-computed tomography (µCT) data)—for a noninvasive, semi-automated 3D segmentation and subsequent comprehensive morphological analysis of the most spatially challenging Choroid Plexus—Hindbrain Choroid Plexus. Using our pipeline on the µCT data of the wild-type (WT) and diverse mutant mouse embryos, we succeeded in the segmentation of the standard Hindbrain Choroid Plexus of the various developmental stages as well as its morphologically impaired forms. Additionally, our pipeline allows users to perform automatically a vast majority of morphological and spatial analyses, which comprehensively describe the Hindbrain Choroid Plexus character in a spatiotemporal manner.

In general, we propose that Ch^O^P-CT, freely available to the academic community, has the tremendous potential to enhance not only general knowledge about the Hindbrain Choroid Plexus ontogenesis and surrounding Choroid Plexus—regulated tissue but primarily can speed up the unbiased identification of the genes indispensable for the proper Hindbrain Choroid Plexus morphogenesis and development once used on the µCT data of the mice deficient in selected genes of interest. All this can ultimately highlight the key genes whose potential deregulation can stand behind not merely the morphologically altered Hindbrain Choroid Plexus but also the dawn of the diseases and syndromes related to the Hindbrain Choroid Plexus and/or surrounding neural tissue. Thus, the Ch^O^P-CT can serve as a convenient tool dramatically boosting the basic as well as applied research of Choroid Plexus.

## Materials and methods

All models, chemicals, machines, and software needed for this study are listed in Additional file 4: Table S1.

### Mouse strains and breeding

All mouse strains used in this project were described previously. Specifically, the generation of the *Cdk13*^tm1a/tm1a^ and *Cdk13*^tm1d/tm1d^ (in this article referred to as *Cdk13* hypomorphic mutant (HM) and *Cdk13* knockout (KO), respectively) was described in reference no. 24. Details on *Tmem107*-/- mice (referred to as *Tmem107* KO in this article) can be found in references no. 26 and 33. All strains used in this article were produced on the C57BL/6 J background.

### Sample preparation

To ensure the sample pool with sufficient biological variability, we collected embryos (5 E13.5 WTs, 5 E15.5 WTs, 5 E17.5 WTs. 3 E13.5 *Cdk13* HMs, 3 E13.5 *Cdk13* KOs, 3 E15.5 *Tmem107* KOs, the summary can be also found in Additional file 4: Table S3) from at least 2 individual mating. The morning after the mating, the females were inspected for vaginal plugs and the noon of the examination day was set as E0.5.

Overall, the pregnant mice were euthanized by the CO_2_ overdose followed by the cervical dislocation and the embryos of the desired genotype (*Cdk13* HMs, *Cdk13* KOs, *Tmem107* KOs or WTs) and stage (E13.5, E15.5 or E17.5) were removed and preserved in ice-cold 1 × Phosphate-Buffered Saline. Then, embryos were fixed for at least 24 h in 4% Paraformaldehyde (P6148, Sigma) and dehydrated using ethanol grade [10%, 30%, 50%, 70%, 80%, 90% EtOH (71250–11001, Penta)]. Before µCT measurement, the samples were stained in a solution containing 1% I_2_ (17570–30250, Penta) in 90% methanol (21210–11000, Penta). Lastly, stained embryos were washed in 30% and 10% EtOH solutions before being subjected to µCT measurements. The duration of dehydration, staining and washing steps varied depending on the specific embryonic stage and the exact times can be found in Additional file 4: Table S2. For in situ hybridization, the dehydrated embryos were cleared by the xylene (601-022-009, Penta) and embedded into the paraffine (HI-00403, Bamed).

### µCT scanning

To immobilize the stained embryos during µCT scanning and minimize motion artefacts, samples were carefully placed in 15 ml Falcon conical centrifuge tubes containing 1% agarose gel (P045, Top-BIO). µCT measurements were conducted using a GE Phoenix v|tome|x L 240 (Waygate Technologies, Baker Hughes Digital Solutions GmbH) laboratory system equipped with a nano-focus X-ray tube with a maximum power of 180 kV/15 W and a high-contrast flat panel detector dynamic 41|100 (number of pixels: 4048 × 4048 px, pixel size 100 μm). The minimum voxel size of the described system is 1 μm. The measurements were acquired with the following settings: an acceleration voltage of 60 kV, an X-ray tube current of 200 µA, and an exposure time of 900 ms. The average volume size of the samples was 1400 pixels × 1400 pixels × 1500 pixels, with an average voxel size of 4 µm. The voxel sizes were variable for different sample sizes and are listed in Additional file 4: Table S3 The tomographic reconstruction was performed using the GE Phoenix datos|× 2.0 3D computed tomography software (Waygate Technologies GmbH Germany). During the reconstruction, the scan optimizer module was used to account for the small and smooth drift of the axis (samples and detector) and focus (X-ray tube) positions.

### Image quality measurements

The contrast in the image was essential to be mentioned to point out the recommended image quality for the successful segmentation by the algorithm. The contrast between Hindbrain Choroid Plexus tissue and its surrounding tissue was computed using the contrast-to-noise ratio formula [19]:

$$contrast \,to\, noise \, ratio= \frac{|{\overline{x} }_{HbChP}-{\overline{x} }_{bg}|}{{\sigma }_{bg}}$$

where ${\overline{x} }_{HbChP}$ is the mean value of Hindbrain Choroid Plexus tissue, and ${\overline{x} }_{bg }\mathrm{and }{\sigma }_{bg}$ are the mean value of the surrounding tissue and the standard deviation of surrounding tissue, respectively. The formula was also used for the contrast-to-noise ratio value computation, where the Hindbrain Choroid Plexus tissue was compared with the image´s background selected outside of the embryo.

### Segmentation

Obtained µCT scans were visualized in VGStudio MAX 2023.1 licensed software (Volume Graphics GmbH, Germany). Subsequently, the slices (transverse plane) containing the head region were extracted and saved as an image stack in.tiff format for further image processing. The segmentation of the Hindbrain Choroid Plexus was implemented in MATLAB 2022b, and a custom function for automatic segmentation was developed and is available on GitHub as a Ch^O^P-CT script. The function requires on its input an image stack, reference fourth ventricle model and a parameter defining the embryonic developmental stage, either E13.5 or E15.5/E17.5. Depending on the specified parameter, the algorithm performs whole Hindbrain Choroid Plexus segmentation for younger samples (E13.5) and partial segmentation, resulting in a mask covering the central part of the Hindbrain Choroid Plexus for older developmental stages. This mask is then exported for manual segmentation of the Hindbrain Choroid Plexus's lateral branches, which are in the end added to the final mask.

### Automatic segmentation by the Ch^O^P-CT script

The image stack was imported into a workspace and converted into a double format. Before the segmentation, the data preprocessing was performed by 3D median filtering along all three dimensions, which resulted in a smoother and less noisy 3D image. Additionally, the data was cropped using a predefined bounding box to reduce the background region and optimize the data size for subsequent processing steps.

### Localisation of the Hindbrain Choroid Plexus

The Hindbrain Choroid Plexus localization in the 3D imaging data was performed by segmentation of the ventricular system with the main focus on the 4th ventricle lying in the hindbrain region. Here, we took advantage of the distinctive characteristics of the ventricular system within the embryonic brain, where it appears as a low-intensity area in the µCT image, as this region is filled up with cerebrospinal fluid. Therefore, thresholding was first applied to the image to extract low-intensity voxels. Secondly, the thresholded image was split into multiple connected components, one of which represented the mask of the 4th ventricle. However, a false connection can be created in the image due to poor contrast between different tissues and the 4th ventricle is connected with other parts of the ventricular system. Hence, the morphological operations were applied before the connected components were computed. The morphological operation led to the separation of the 4th ventricle from the ventricular system mask. These connected regions were then registered with a reference mask of the 4th ventricle which was downloaded from the atlas presented by Wong et al., 2012 [20]. The registration was achieved by firstly converting both regions which are being compared into point clouds followed by the application of the iterative closest point algorithm, which can be found as a build function in MATLAB called pcregistericp. The registration process was assessed using the Hausdorff distance to select the region with the highest similarity to the reference mask, resulting in our primary mask for the 4th ventricle. Thirdly, the final mask of the ventricular system was enhanced by active contours applied on the complement of the grayscale image for smoothing and refinement.

### Hindbrain Choroid Plexus segmentation

Firstly, the morphological closing was applied to the mask of the ventricular system. This image processing technique was used to fill in gaps in binary images resulting in the successful incorporation of Hindbrain Choroid Plexus voxels into the targeted masked region. Subsequently, the closed mask was subtracted from the original to generate a mask containing a Hindbrain Choroid Plexus. Voxels of other structures were also included in the mask along the light areas representing the Hindbrain Choroid Plexus. Therefore, the high-intensity voxels were extracted from the region outlined by a generated mask to retrieve the demanded voxels. Finally, all outliers not representing the Hindbrain Choroid Plexus were removed by the selection of the largest connected components in the primary mask.

### E13.5 Hindbrain Choroid Plexus segmentation

The E13.5 Hindbrain Choroid Plexus does not extend into the lateral parts of the 4th ventricle where the proposed automatic segmentation meets its challenges. Therefore, the fully automatic segmentation was developed and is further described. The generated primary mask of the E13.5 Hindbrain Choroid Plexus served to localize its partial volume and to extract information describing this complex structure to achieve segmentation of the entire volume in µCT data. Firstly, a local range analysis was performed on a grayscale dataset, resulting in the generation of an image representing the range values. Subsequently, the mean range within the region defined by the primary mask was computed and used as a threshold for the corresponding image. Secondly, connected regions intersecting with the primary mask were selected in the resulting image, followed by the application of morphological closing. The Hindbrain Choroid Plexus was extracted from the area masked by selected regions by removing the low-intensity voxels. Thirdly, the mask of the Hindbrain Choroid Plexus was altered by morphological opening to exclude voxels belonging to other tissue, and again the largest connected component was selected. Lastly, the active contour model was applied to achieve the segmentation of the entire Hindbrain Choroid Plexus volume, incorporating the mask obtained from the preceding steps. For enhanced visualization, the mask with its appropriate image data of the selected sample was exported as image stacks in.tiff format from MATLAB and consequently imported to Avizo 9.5 software. Within Avizo, the mask representing the Hindbrain Choroid Plexus was converted into a.stl model and subsequently exported for further analysis or visualization purposes.

### E15.5 and E17.5 Hindbrain Choroid Plexus segmentation

A primary mask was not further processed by the presented segmentation algorithm in MATLAB. Developmentally older embryos have almost fully developed Hindbrain Choroid Plexus which extends into lateral parts of the 4th ventricle where the contrast between its structure and the surrounding tissue is not sufficient to proceed with automatic segmentation. Hence, the primary mask is exported from MATLAB as an image stack in.tiff format, indicating the need for manual segmentation in these cases.

### Manual segmentation

The segmentation of lateral Choroidal branches, which are prolonged to the lateral recesses of the 4th ventricle, was challenging for older embryos, namely E15.5 and E17.5. Hence, these branches were not successfully included in the automatic segmentation used for E13.5 species, and additional manual segmentation was necessary. The manual segmentation was carried out using Avizo 9.5 software (Thermo Fisher Scientific) by importing the exported automatically segmented Hindbrain Choroid Plexus primary mask. The semi-automatic segmentation was performed, and the final 3D model was exported as the 3D.stl file.

### 3D mean model

The mean model of the Hindbrain Choroid Plexus was generated for each available embryonic developmental stage by calculating the mean of the 5 WT 3D models. The registration was achieved by the manual alignment followed by the best-fit registration in the professional software VGStudio MAX 2023.1. In the same software, the computation of the mean model was performed by the golden surface module resulting in the mean model from the 5 inputs.

### Automatic morphological analysis using Ch^O^P-CT script

#### a. Volume analysis

To calculate the volume, the number of voxels within the 3D segmented model was multiplied by the voxel size:

$$Volume = Number \, Of \, Voxels * Voxel \, Size (mm3)$$

#### b. Surface area

The Crofton formula was used to determine the area of the Hindbrain Choroid Plexus binary model effectively by measuring the distance around the boundary of the region. The Crofton method employs a technique that counts the number of model edges intercepted by a set of isotropic test lines. This method yields an approximation of the surface area S of the 3D object based on this relation:

$$S\left(X\right)\simeq 4{\sum }_{k}\frac{{c}_{k}}{{\lambda }_{k}}\chi \left(X\cap {L}_{k}\right) \left(m{m}^{3}\right)$$

where L_k_ is a group of 3D discrete lines parallel to direction k, c_k_ is the discretization weight associated with 3D direction k, and λ_k_ is the density of discrete lines in direction k. The density of a line is calculated by dividing the distance between neighbouring voxels in direction k by the volume of a single voxel. Weight c_k_ is equal to 1⁄3. Intercept counts are calculated using run-length encoding. The run-length encoding collects all pixels on a single line into one element. This metric was calculated using the function regionprops3 in MATLAB.

#### c. Outgrowth angle

The estimation of the ventral Hindbrain Choroid Plexus outgrowth angle was made by determining the angle between the direction vector of the Hindbrain Choroid Plexus and a posteriorly placed frontal plane within the ventricle. To make this calculation, a 3D model of the Hindbrain Choroid Plexus was utilized, and eigenvectors were used to determine the orientation of the fitted ellipsoid. The eigenvalues were calculated prior to determining the eigenvectors using the equation:

$$|A-\lambda \cdot I|=0$$

where A is a matrix, λ is the eigenvalue, and I is the identity matrix. The eigenvalues are given in descending order based on the variability. Next, the eigenvectors were computed using the formula:

$$A\cdot {v}_{k}={\lambda }_{k}\cdot {v}_{k}$$

where v_k_ is the k-th eigenvector and λ_k_ is the k-th eigenvalue. Finally, the angle was computed using the first eigenvector v_1_, which defines the direction of the model, and the vector of the sagittal plane (X-axis), which is equal to u = ⟨1, 0, 0⟩. The angle was then computed with the following formula:

$$angle\, = \,\cos^{- 1} \left( {\frac{{\overrightarrow {u} \, \cdot \,\overrightarrow {{v}_{1} } }}{{\left\| u \right\|\,\left\| {v}_{1}  \right\|}}} \right)\, * \,\frac{180}{\pi }\,\left( {\circ} \right)$$

#### d. Generalized procrustes analysis using landmarking

The 3D Hindbrain Choroid Plexus models were compared by selecting a suitable set of landmarks and analyzing them using Generalized Procrustes Analysis. A landmark refers to the point that corresponds to the same anatomical or mathematical location across the different samples. By selecting a landmark for each model at the same location, the variation in the position of the landmark can be utilized to compare the models. Using the Markups module in Slicer 5.2.1 software, 10 landmark points were manually chosen from each model. The Generalized Procrustes Analysis was conducted, where each model was compared to a determined mean model by comparing pairs of landmarks. Procrustes distances, which are the minimum distances between two points, were used in Generalized Procrustes Analysis. The Procrustes distance was computed as the sum of squared differences between sample landmarks and mean model landmarks. The Generalized Procrustes Analysis method aligned models and removed the effects of translation, scale, and rotation. Briefly, the Generalized Procrustes Analysis computation involves four steps: (1) the initial mean model is estimated by selecting the first model landmarks; (2) the remaining models’ landmarks are aligned to the mean model landmarks; (3) the estimate of the mean model is recalculated from the aligned models; and (4) if there is a difference between the estimated mean model and the initial mean model, the algorithm returns to step two until the model no longer changes significantly in further iterations. Finally, landmarks were transformed using the derived mean model, and the Procrustes distances were computed. This computation was performed for each sample with the mean model. The Generalized Procrustes Analysis analysis was implemented in MATLAB using a modified algorithm created by Pulak Purkait, 2020. Furthermore, the mean model from segmented 3D models was used directly for Generalized Procrustes Analysis in analyzing mutant variations, eliminating the need for iterative optimization within the algorithm. In the case of *Tmem107* KOs analysis, only a procrusion distance data from specific landmarks (LM 5,6, 7, 8, 10, 11) were selected and further analyzed.

#### e. The Hindbrain Choroid Plexus proportional analysis

The proportion of the Hindbrain Choroid Plexus in the 4th ventricle was calculated using their segmented models by dividing the Choroid Plexus volume by the sum of both models (Hindbrain Choroid Plexus, 4th ventricle).

### Manual morphological analysis

#### a. Analysis of the lateral expansion of the Hindbrain Choroid Plexus

To measure the length of the lateral protrusions of the Hindbrain Choroid Plexus branches within the 4th ventricle, the measurement process was initiated from the Hindbrain Choroid Plexus centre and proceeded towards the end of the ventricle. For determining the length of the main Hindbrain Choroid Plexus body, the total length of each branch was subtracted from the overall length of the ventricle. The measurement was done in VGStudio MAX 2023.1 in the transverse plane.

#### b. Left–right symmetry analysis using the branch´s length and outgrowth angle

The length and angle of the left and right branches were measured manually in VGStudio MAX 2023.1 in the transverse plane. The length was evaluated from the Hindbrain Choroid Plexus centre, which was positioned on the brain's central axis in the transverse plane until the end of the branches, which are laterally prolonged into the lateral recesses of the 4th ventricle. Both the left and right angles between the brain's central axis and the relevant red line were measured.

#### c. Hindbrain Choroid Plexus distribution along the rostrocaudal axis

To determine the proportional volume of the rostral part of the Hindbrain Choroid Plexus relative to the caudal part, a manual selection process was employed to select the respective segments of the model. The upper part was manually outlined, and its volume was calculated. Subsequently, the volume of the lower part was determined by subtracting the volume of the upper part from the total volume of the Hindbrain Choroid Plexus. The manual selection was performed using Avizo 9.5 software.

### Third-party µCT scans

Publicly available µ-CT scans were downloaded from The International Mouse Phenotyping Consortium webpage. More specifically, the µCT scans of WT E15.5 female embryo were downloaded in the best quality available (pixel size: 13.45 µm).

### Statistics

All presented graphs were generated by GraphPad Prism 9 software. The presented data were measured within 3 particular groups—WT (E13.5—n = 5, E15.5—n = 5, E17.5—n = 5), *Cdk13* mutants (E13.5 *Cdk13* HM—n = 3, E13.5 *Cdk13* KO—n = 3) and *Tmem107* mutants (E15.5 *Tmem107* KO—n = 3). The significance was measured by the One-way ANOVA, Two-way ANOVA or unpaired Student´s t-test, The data are presented in columns showing the mean with standard deviation. The specific type of used statistical test is always stated in the figure legend of the individual graphs. Of note, due to the uneven sample size for the analysis of the potential breakage of left/right symmetry and caudal/rostral proportions within the *Cdk13* and *Tmem107* mutants embryos, the mean, standard deviations and number of replicates calculated ahead were used for the Two-way ANOVA tests.

### In situ hybridization

To detect the transcripts of candidate genes within the WT as well as mutant tissues, the paraffin-embedded mouse embryos of various developmental stages and genotypes were sectioned for 5 µm using a microtome (RM2145, Leica). Then, slices were deparaffinized and dehydrated by the absolute EtOH, followed by the H_2_O_2_ (SIAL95294, Merck) treatment to block endogenous peroxidase. After the rinse in the distilled H_2_O, the slices were boiled in RNAscope^®^ Target Retrieval Reagent (322000, ACD Bio) at 100 °C for 10 min. This was followed by the protease treatment (322331, ACD Bio) and probe hybridization step for 2 h in a hybridization oven (HybEZ^™^ II, ACD Bio) at 40 °C. Individual probes are listed in the Additional file 4: Table S1. The gene expression of the targeted genes was detected by the RNAscope^®^ Multiplex Fluorescent v2 Assay kit (323110, ACD Bio, USA) and TSA Plus Cyanine 3 (1:1000; NEL744001KT, Perkin Elmer) according to the manufacturer´s protocol. The nuclei were stained by DAPI (323108, ACD Bio) and slides were mounted by the FluoroshieldTM (F6059, Sigma-Aldrich). Images were acquired using the Zeiss LSM 800 microscope and exported via the Zen Blue software.

## Results

### The Ch^O^P-CT tool in a nutshell

**Fig. 1:**
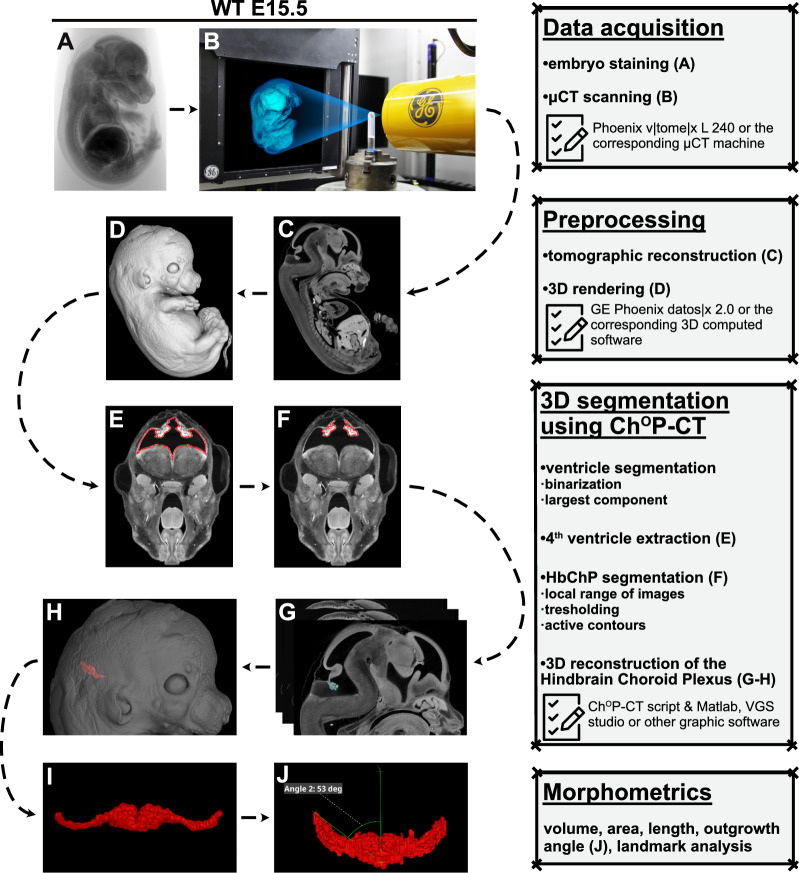
The Ch^O^P-CT tool in a nutshell. Summary of the Ch^O^P-CT protocol. Shortly, the µCT scanning of the stained embryo is followed by the tomography reconstruction and 3D rendering. Then, the brain ventricular system is segmented. After this, the Ch^O^P-CT script operates with the extracted 4th ventricle, where the Hindbrain Choroid Plexus is located. The final Hindbrain Choroid Plexus 3D model is obtained by the application of the active contour and subsequent 3D modelling. The obtained Hindbrain Choroid Plexus 3D model is then used for the morphological analysis. For further details, please, see the section Materials and Methods. Of note, WT E15.5 embryo was here used for the demonstration of the Ch^O^P-CT workflow. *3D* three dimensional, *µCT* X-ray micro-computed tomography, *E* embryonic day, *Ch*^*O*^*P-CT* Ch^O^Pping the Ch^O^roid Plexus out of the µCT data, *WT* wild-type

To visualize and morphologically assess the developing Hindbrain Choroid Plexus tissue occupying the 4th ventricle, we developed the tool Ch^O^P-CT—a publicly available pipeline for Hindbrain Choroid Plexus segmentation and further morphometric analysis (Fig. 1). Shortly, this MATLAB-based script is operating with the µCT scans of the whole embryos, on which the Ch^O^P-CT tool can localise and segment the Hindbrain Choroid Plexus tissue (whole or its central part, further discussed in the next chapter) within the 4th ventricle. Using the obtained Hindbrain Choroid Plexus 3D model, the pipeline enables the user to further perform the set of measurements (e.g. assessment of the Hindbrain Choroid Plexus volume, area, landmark analysis etc.). On top of this, we also provide a guide for the additional, manually performed morphometric of the Hindbrain Choroid Plexus (e.g. length or angle of the individual Hindbrain Choroid Plexus branches etc.). Together with the automatically measured set of parameters, this criterion panel can provide a complex picture of the actual morphometric status of the investigated Hindbrain Choroid Plexus tissue.

### 3D visualization of the developing murine Hindbrain Choroid Plexus

**Fig. 2:**
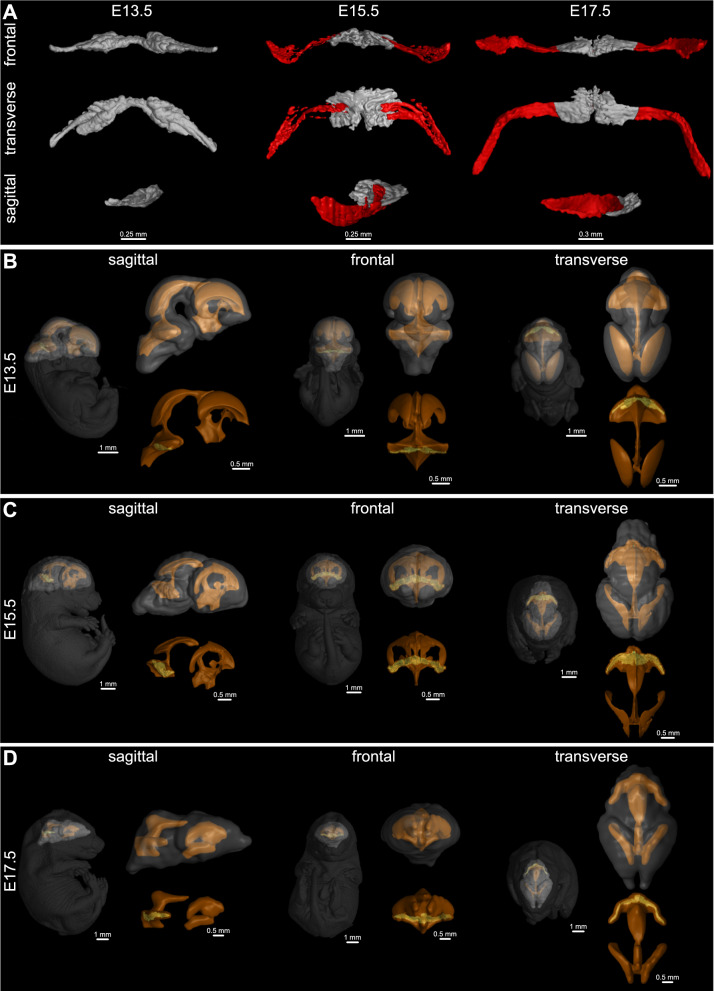
3D visualization of the developing murine Hindbrain Choroid Plexus in WT embryos. **A** Frontal (top horizontal panel), transverse (middle horizontal panel) and sagittal (bottom horizontal panel) views of gross anatomy and microscopic structure of WT Hindbrain Choroid Plexus of E13.5 (left vertical panel), E15.5 (middle vertical panel) and E17.5 (right vertical panel). These 3D models were produced by the Ch^O^P-CT tool (grey area of the 3D model), which was followed by the manual segmentation of the lateral segments of Hindbrain Choroid Plexus (red area of the 3D model). Scale bar: left/middle vertical panels: 0.25 mm, the right vertical panel: 0.3 mm. **B**–**D** Visualization of the scanned whole embryos (E13.5—**B**, E15.5—**C**, E17.5—**D**) together with the manually segmented brain (grey) and ventricular system (yellow). For the spatial context, the 3D Hindbrain Choroid Plexus model (green) obtained by Ch^O^P-CT script and manual segmentation (stages E15.5 and E17.5) is visualized within the ventricular system (yellow). The left vertical panel always represents the sagittal view, while the middle vertical panel display the segmented parts in the frontal view. The right vertical panel demonstrates individual body parts in the transverse view. The interactive pdfs presenting the position of all developmentally diverse Hindbrain Choroid Plexuses within the corresponding ventricular system and head are attached (Additional files 1, 2, 3). *3D* three dimensional, *µCT* X-ray micro-computed tomography, *E* embryonic day, *Ch*^*O*^*P-CT* Ch^O^Pping the Ch^O^roid Plexus out of the µCT data, *WT* wild-type

To fully disclose the whole potential of the Ch^O^P-CT script, we challenged this tool with multiple datasets of the developmentally diverse stages of the mouse embryos, which were scanned by our laboratory. The Ch^O^P-CT tool was able to provide a fully segmented Hindbrain Choroid Plexus at the E13.5 (Fig. 2A, left panel, grey). However, as the morphological complexity of the Hindbrain Choroid Plexus increases along the developing timeline, our tool was able to segment just the central part of the E15.5 or older Hindbrain Choroid Plexus tissues within the µCT scans (Additional file 4: Fig. S1A, grey) and the lateral protrusion of the Hindbrain Choroid Plexuss has to be segmented manually (Additional file 4: Fig. S1A, red). Thus, the final Hindbrain Choroid Plexus 3D models of E15.5 and E17.5 are composed of the automatically segmented central area of the Hindbrain Choroid Plexus (Fig. 2A grey, middle and right panels) and manually chopped Hindbrain Choroid Plexus lateral protrusions (Fig. 2A, red, middle and right panels), which was executed by the fine-tuning of the manually segmented Hindbrain Choroid Plexus mask (for further details, please, see the section Material and methods).

To completely utilize the information and technological capacity achieved with the usage of the µCT data, we further manually segmented the whole ventricular system and surrounding brain of all analysed developmental stages (Fig. 2B–D). Together with the Hindbrain Choroid Plexus 3D models, these visualizations were used for the generation of interactive.pdf files (Additional files 1, 2, 3), which provide a reader with the spatial information of the Hindbrain Choroid Plexus in the context of the whole central nervous system.

After the establishment of the whole process, we wanted to test the Ch^O^P-CT tool on the external type of data. Thus, we explored the µCT scans, which are publicly available on the International Mouse Phenotyping Consortium webpage and have already been used for segmentation analyses [21, 22]. However, due to the low quality of the datasets available in this repository, our tool failed to segment even the central part of the Hindbrain Choroid Plexus in the details and included the additional surrounding neural tissues (Additional file 4: Fig. S1B, red). Therefore, we chose to continue just with our µCT data where the voxel size was below 10 µm, the contrast-to-noise ratio [19] was, on average, 17 dB and the contrast between Hindbrain Choroid Plexus tissue and surrounding attached tissue was set to 8 dB using the contrast-to noise ratio formula. Furthermore, we optimized scanning parameters for biological samples, including lower voltage, sufficient projections, and minimized spot drift. This optimization enabled comprehensive Hindbrain Choroid Plexus segmentation for the subsequent morphological assessment.

### Spatiotemporal morphological assessment of the developing Hindbrain Choroid Plexus

**Fig. 3:**
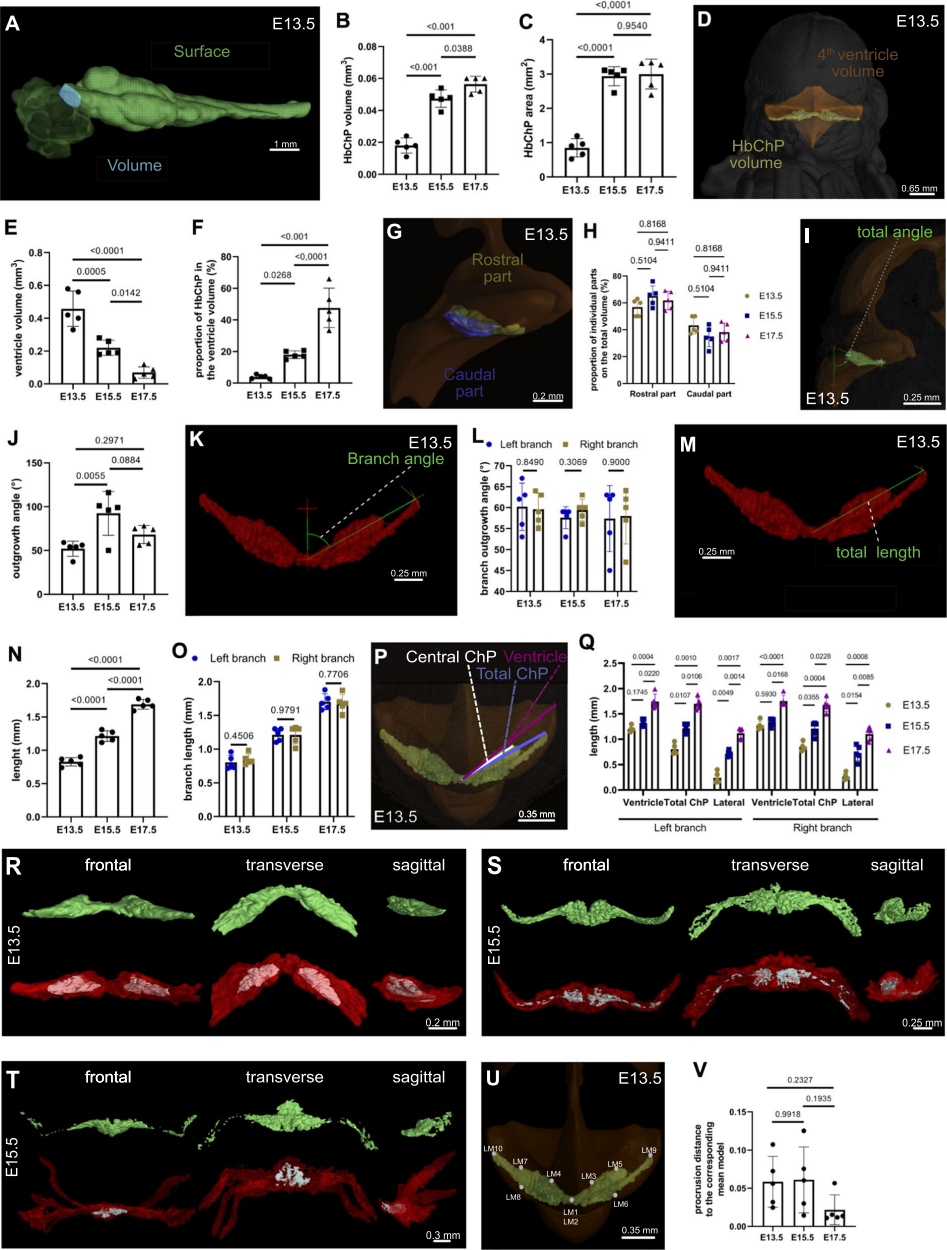
Spatiotemporal morphological assessment of the developing Hindbrain Choroid Plexus. **A** The illustration of the Ch^O^P-CT-based measurement method of the total volume (cyan) and surface (green) is visualized on the frontal view of E13.5 Hindbrain Choroid Plexus. Scale bar: 1 mm. **B** Quantification of the total volume differences between the WT Hindbrain Choroid Plexuses of E13.5, E15.5 and E17.5 demonstrates a statistically significant increase in the total Hindbrain Choroid Plexus volume along the analyzed developing timeline. Note for **B**, **C**, **E**, **F**, **J**, **N**, **O**, **V**—the number of biological replicates within the particular group, n = 5, also indicated in the graph,. Error bars indicate the standard deviation, p-values—one-way ANOVA with Turkey´s multiple comparison test. Note for **H**, **L**, **O**, **Q**—the number of biological replicates within the particular group, n = 5, also indicated in the graph. Error bars indicate the standard deviation, p-values—two-way ANOVA with Turkey´s multiple comparison test. **C** Quantification of the surface area shifts between the WT Hindbrain Choroid Plexuses of E13.5, E15.5 and E17.5 displaying the statistically significant increase just between the first two analyzed samples. **D** Representation of the total Hindbrain Choroid Plexus volume (green) and the ventricle volume (yellow) measurement is visualized within the frontally positioned E13.5 embryo, which was stained by Lugol´s solution prior to µCT scanning. The data acquisition for this analysis was performed by the Ch^O^P-CT tool. Scale bar: 0.65 mm. **E** Graphical representation of the gradual expansion of the 4th ventricle volume, which was observed every two days within our monitored period of embryonic development (from E13.5 to E17.5). **F** Quantification of the shift in the proportional test of the Hindbrain Choroid Plexus volume in the total volume of the 4th ventricle between the WT E13.5, E15.5 and E17.5. **G** Sagittally presented a 3D reconstructed ventricular system and 3D model of the E13.5 Hindbrain Choroid Plexus illustrating the manual outlining of the rostral (green) and caudal (blue) parts of the Hindbrain Choroid Plexus used for the volume quantification of these Hindbrain Choroid Plexus parts. Scale bar: 0.2 mm. **H** No significant shift was observed in the volume proportion of the rostral and caudal part of the WT Hindbrain Choroid Plexuses of E13.5, E15.5 and E17.5. **I** The illustration of the Ch^O^P-CT-based measurement of the Hindbrain Choroid Plexus outgrowth angle into the 4th ventricle visualized on the sagittally positioned E13.5 3D Hindbrain Choroid Plexus and reconstructed brain ventricular system (yellow). Scale bar: 0.25 mm. **J** Quantification of the differences in Hindbrain Choroid Plexus outgrowth angle between the WT E13.5, E15.5 and E17.5, showing the significant inclination of the E15.5 Hindbrain Choroid Plexus to the above-lying cerebellum compared to other (E13.5 and E17.5) analyzed samples. **K** Transversally positioned 3D model of the E13.5 Hindbrain Choroid Plexus illustrating the manual measurement of the outgrowth angle of the individual (left and right) Hindbrain Choroid Plexus branches. Scale bar: 0.25 mm. **L** No disruption of the left–right symmetry between the WT Hindbrain Choroid Plexuses of E13.5, E15.5 and E17.5 in terms of outgrowth angle was detected by the quantifications of the outgrowth angle parameter. **M** Transversally positioned 3D model of the E13.5 Hindbrain Choroid Plexus model illustrating the manual measurement of the total length of the individual (left and right) Hindbrain Choroid Plexus branches. Scale bar: 0.25 mm. **N** Quantification of the Hindbrain Choroid Plexus length differences between the WT Hindbrain Choroid Plexuses of E13.5, E15.5 and E17.5 presenting the gradual expansion of the Hindbrain Choroid Plexus into the ventricular space. **O** Quantification of the variations in left and right Hindbrain Choroid Plexus branch length between the WT Hindbrain Choroid Plexuses of E13.5, E15.5 and E17.5. This manually measured analysis confirmed no breakage of the left–right symmetry in this morphological parameter. **P** The illustration of the length measurement of the ventricle (pink), the whole Hindbrain Choroid Plexus (blue) and the central Hindbrain Choroid Plexus part (white) using the E13.5 Hindbrain Choroid Plexus 3D model positioned in the segmented dorsal ventricular system. These body parts are presented within the embryo in the transverse plane. Scale bar: 0.35 mm. **Q** Quantification of the differences in the ventricle length, total Hindbrain Choroid Plexus length as well as the length of the central Hindbrain Choroid Plexus between the WT Hindbrain Choroid Plexuses of E13.5, E15.5 and E17.5 displaying the increasing proportion of lateral Hindbrain Choroid Plexus segments within the lateral recesses of the 4th ventricle as the total Choroid Plexus length is gradually increasing but the length of the Hindbrain Choroid Plexus central part does not. The analysis showed no differences between the left and right branches. **R**–**T** Frontal (left vertical panel), transverse (middle vertical panel) as well as sagittal (right vertical panel) views of the mean (green), minimal (grey) as well as maximal (red) models of the Hindbrain Choroid Plexus of E13.5 (**S**), E15.5 (**T**) and E17.5 (**U**). Scale bars can be found within the figures. **U** The illustration of the position of the landmarks within the E13.5 mean model of Hindbrain Choroid Plexus, which is positioned transversally. Scale bar: 0.35 mm. **V** Higher shape variability was detected within the group of E13.5 and E15.5 Hindbrain Choroid Plexuses compared to the E17.5 Hindbrain Choroid Plexus group as these two groups display greater procrusion distances between the landmarks placed within the individual Hindbrain Choroid Plexus 3D model and corresponding 3D mean models. Scale bars can be found in the figures. 3D three dimensional, *µCT* X-ray micro-computed tomography, *E* embryonic day, *Ch*^*O*^*P-CT* Ch^O^Pping the Ch^O^roid Plexus out of the µCT data, *WT* wild-type

**Fig. 4:**
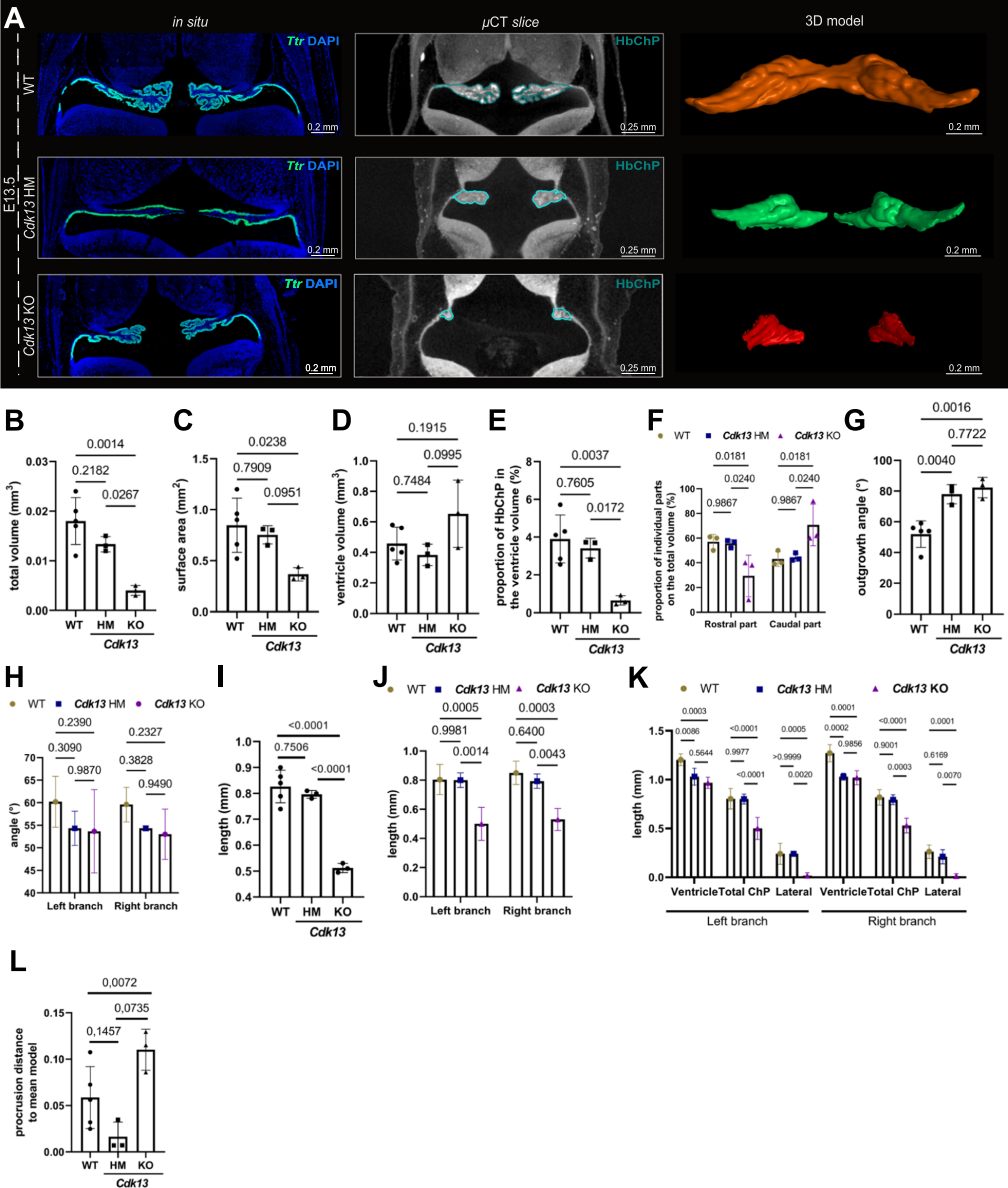
Detection of the morphologically altered Hindbrain Choroid Plexus of *Cdk13* mutant embryos. **A** The visualization of the Hindbrain Choroid Plexus within the E13.5 WT (top horizontal panel), *Cdk13* HM (middle horizontal panel) as well as *Cdk13* KO (bottom horizontal panel) by the in situ analysis of the Hindbrain Choroid Plexus epithelial marker—*Ttr* (left vertical panel), single µCT slice (middle vertical panel) as well as 3D model (right vertical panel). Individual scale bars can be found within the images. **B** Hindbrain Choroid Plexus volume reduction was detected within the E13.5 *Cdk13* KO mouse embryos compared to the WT as well as *Cdk13* HM. Note for **B**, **C**, **D**, **E**, **G**, **I**, **L**—Individual data points representing biological replicates (WT—n = 5, *Cdk13* HM—n = 3, *Cdk13* KO—n = 3) and standard deviation are presented; statistical differences were analyzed by one-way ANOVA with Turkey´s multiple comparison test, and individual p values are indicated. Note for **F**, **H**, **J**, **K**—The data points visualized together with standard deviations as the error bars within the graphs represent the mean of measured parameters from WT—n = 5, *Cdk13* HM—n = 3, *Cdk13* KO—n = 3, p-values = two-way ANOVA with Turkey´s multiple comparison test **C** Quantification of the Hindbrain Choroid Plexus area differences between the E13.5 Hindbrain Choroid Plexus of WT, *Cdk13* HM and *Cdk13* KO, displaying the trend of decrease in this morphological parameter within the *Cdk13* HM and primarily *Cdk13* KO embryos. **D** An increase was detected in the 4th ventricle volume of *Cdk13* KO compared to WT and *Cdk13* HM individuals. **E** A strong reduction of the Hindbrain Choroid Plexus volume proportion in the 4th ventricle volume was observed within the *Cdk13* KOs compared to the WT as well as *Cdk13* HM embryos. **F** The disbalance in the Hindbrain Choroid Plexus outgrowth along the rostrocaudal axis was detected within the *Cdk13* KO embryos comparing to the WT and *Cdk13* HM **G**–**H** Quantification of the Hindbrain Choroid Plexus outgrowth angle differences between the E13.5 Hindbrain Choroid Plexus of WT, *Cdk13* HM and *Cdk13* KO, displaying the trend of increase in *Cdk13* HM as well as *Cdk13* KO embryos (**G**). However, no differences between the right and left Hindbrain Choroid Plexus branch outgrowth angles were detected (**H**). **I**–**J** The decrease in the Hindbrain Choroid Plexus length was observed within the *Cdk13* KO groups compared to the WTs as well as *Cdk13* HMs (**I**), while no disruption in the left–right symmetry was uncovered in this morphological parameter (**J**). **K** Quantification of the ventricle length; total Hindbrain Choroid Plexus length as well as the length of the central Hindbrain Choroid Plexus differences between the E13.5 WTs, *Cdk13* HMs and *Cdk13* KOs presenting the absence of the lateral parts of Hindbrain Choroid Plexus within the lateral recesses of the 4th ventricle of *Cdk13* KOs, while the other measured parameters remain unchanged. **L** Quantification of the landmark analysis determined the large shape range between all analyzed samples (E13.5 *Cdk13* HM and *Cdk13* KO) compared to the corresponding WTs. More specifically, the low procrusion distance of landmarks placed within the *Cdk13* HM was detected, whereas this parameter is significantly higher within the *Cdk13* KO group. *3D* three dimensional, *µCT* X-ray micro-computed tomography, *E* embryonic day, *KO* knockout, *HM* hypomorphic mutant, *WT* wild-type

**Fig. 5:**
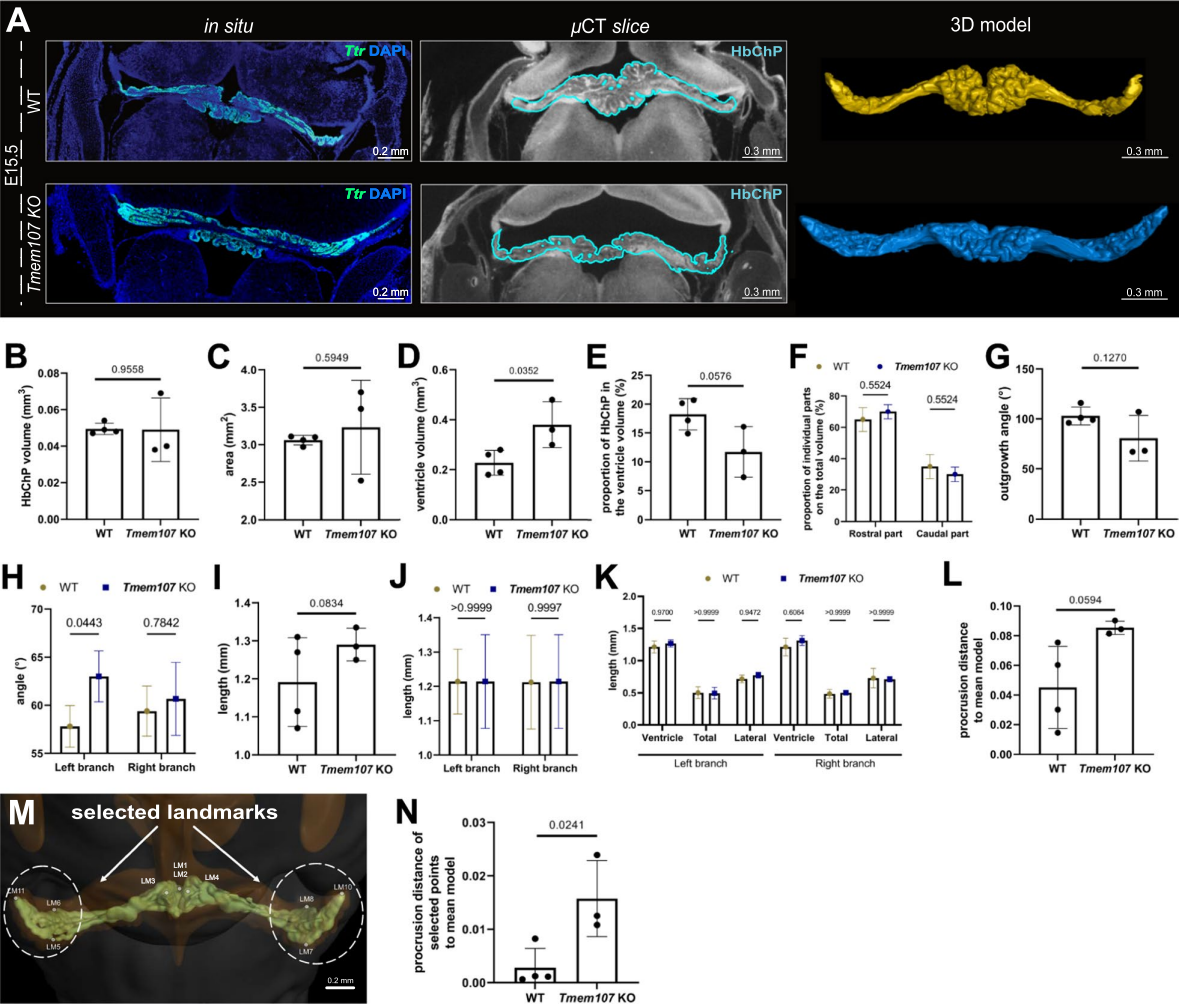
Detection of the morphologically altered Hindbrain Choroid Plexus within the *Tmem107* mutant embryos. **A** The visualization of the Hindbrain Choroid Plexus within the E15.5 WT (top horizontal panel) and *Tmem107* KO (bottom horizontal panel) by the in situ analysis of the Hindbrain Choroid Plexus epithelial marker—*Ttr* (left vertical panel), single µCT slice (middle vertical panel) as well as 3D model (right vertical panel). Individual scale bars can be found within the images. **B**–**C** No statistically significant changes in the Hindbrain Choroid Plexus volume (**B**) or area (**C**) were detected by the analyses of E15.5 WTs and *Tmem107* KOs. Note for **B**, **C**, **D**, **E**, **G**, **I**, **L**, **N**—the number of biological replicates within the particular group is indicated in the graphs, (WT—n = 5, *Tmem107* KO—n = 3). Error bars indicate the standard deviation, p-values—unpaired two-tailed Student´s t-test. Note for **F**, **H**, **J**, **K**—The data points visualized together with standard deviations as the error bars within the graphs represent the mean of measured parameters from WT—n = 5, *Tmem107* KO—n = 3, p = two-way ANOVA with Turkey´s multiple comparison test **D** An increase in the 4th ventricle volume was detected in *Tmem107* KO compared to WT individuals. **E** No significant shift in the Hindbrain Choroid Plexus proportion in the 4th ventricle volume was observed analyzing the WT and *Tmem107* KOs. **F** No statistically significant changes in the volume of the rostral and caudal part of E15.5 WTs and *Tmem107* KOs were observed. **G**, **H** No statistically significant change in total outgrowth angle (**G**) was detected during the analysis of E15.5 Hindbrain Choroid Plexus WTs vs. *Tmem107* KOs. Nonetheless, the left–right breakage symmetry was observed within this morphological parameter (**H**), **I**, **J** No statistically significant change in total length was detected during the analysis of E15.5 Hindbrain Choroid Plexus WTs vs. *Tmem107* KOs (**I**), Additionally, no the left–right breakage symmetry was observed within this morphological parameter (**J**). **K** Quantification of the differences in the ventricle length, total Hindbrain Choroid Plexus length as well as the length of the central Hindbrain Choroid Plexus between the E15.5 WTs and *Tmem107* KOs determining the slight increase in the length of the lateral segments of the Hindbrain Choroid Plexus, which are protruding into the lateral recesses of the 4th ventricle in *Tmem107* KOs, compared to the WT littermates. **L** Quantification of the whole landmark analysis displays no significant shape change between Hindbrain Choroid Plexus E15.5 WTs and *Tmem107* KO. **M** The illustration of the selected landmarks for the specific Landmark analysis for shape analysis of *Tmem107* KOs compared to corresponding WTs. Landmarks are placed within the frontally positioned 3D model of E15.5 WT Hindbrain Choroid Plexus. Scale bar: 0.2 mm. **N** Quantification of the protrusion distance of the selected landmark presenting significant shape change within the lateral segments of the Hindbrain Choroid Plexus comparing E15.5 WTs and *Tmem107* KO. *3D* three dimensional, *µCT* X-ray micro-computed tomography, *E* embryonic day, *I* knockout, *WT* wild-type

Next, we designed the broad panel of main parameters, which serves for the morphological and spatial characterization of the Hindbrain Choroid Plexus. Such an array of the quantitative criteria has a great potential to not only uncover the changes occurring along the developing timeline (Fig. 3) but also to detect the Hindbrain Choroid Plexus morphological alteration of the various mutant embryos as demonstrated later (Figs. 4, 5).

In more depth, we have divided this panel into 4 main categories: (a) Hindbrain Choroid Plexus volume and area analysis (Fig. 3A–H), (b) Hindbrain Choroid Plexus outgrowth angle analysis (Fig. 3I–L), (c) Hindbrain Choroid Plexus length analysis (Fig. 3M–Q) and (d) Shape analysis of Hindbrain Choroid Plexus (Fig. 3U, V). To properly describe the variability of the developing Hindbrain Choroid Plexus, we segmented and evaluated 5 Hindbrain Choroid Plexuses from WT mice for each selected developmental stage (E13.5, E15.5, E17.5) (Additional file 4: Fig. S2A–O).

Within the first category, we first performed the archetypal morphometrics used in the Choroid Plexus field [13–15]—volume (Fig. 3A, blue) and area (Fig. 3A, green) analyses. These Hindbrain Choroid Plexus features are strongly associated with the rate of cerebrospinal fluid production. Indeed, we detected a gradual increase in the Hindbrain Choroid Plexus volume (Fig. 3B) as well as the area **(**Fig. 3C) along the analyzed developmental timeline. In agreement with others [23], we observed a progressive decrease of the 4th ventricle volume (Fig. 3D, E), which, together with the above-mentioned expanding Hindbrain Choroid Plexus resulted in the extreme increase of the Hindbrain Choroid Plexus proportion within the 4th ventricle (Fig. 3D, F). Next, considering the current general knowledge about the Hindbrain Choroid Plexus epithelium regionalization along the rostrocaudal axis [2] we also examined potential differences in developing time windows between rostral (Fig. 3G, green) and caudal (Fig. 3G, blue) Hindbrain Choroid Plexus segments during the embryogenesis. Nonetheless, we did not observe a shift in the volume proportion of any above-mentioned parts in the total Hindbrain Choroid Plexus volume along the monitored period of embryogenesis (Fig. 3H).

Another crucial characteristic enabling the Hindbrain Choroid Plexus tissue to monitor and regulate a wide range of adjacent regions of the developing neural tube is its position and projection within the 4th ventricle [17, 18]. Firstly, we determined the total Hindbrain Choroid Plexus outgrowth angle (Fig. 3I) revealing the Hindbrain Choroid Plexus position shift within the developing 4th brain cavity, from the physical inclination to the above-lying cerebellum (E15.5) to the more central cavity projection of the Hindbrain Choroid Plexus (E13.5 and E17.5) (Fig. 3J). To catch the potential left–right asymmetry in the Hindbrain Choroid Plexus development, we decided to assess this parameter further, focusing separately on the angle of the right and left branches of the Hindbrain Choroid Plexus (Fig. 3K). This analysis pointed to the symmetry maintenance during the Hindbrain Choroid Plexus extension into the 4th ventricle (Fig. 3L).

The projection length of the Hindbrain Choroid Plexus into the 4th cavity is another key morphological category, which also predicates the left–right symmetry/asymmetry character of the developing Hindbrain Choroid Plexus (Fig. 3M). Here, we detected a gradual increase in the total length of the Hindbrain Choroid Plexus every two days of the mouse embryonic development (Fig. 3N) without any breaking of the left–right symmetry (Fig. 3O). Additionally, we aimed to determine the expansion of the lateral segments of the Hindbrain Choroid Plexus into the lateral recesses of the 4th ventricle in time. To target this, we measured the total length of the ventricle (Fig. 3P, pink) as well as the Hindbrain Choroid Plexus (Fig. 3P, blue), along with the length of the Hindbrain Choroid Plexus central part (Fig. 3P, white) and we observed the increasing prevalence of the lateral segments in the total length of the analysed tissue (Fig. 3Q). Noteworthy, we also did not observe any disbalance between the left or right Hindbrain Choroid Plexus in any of the analyzed parameters (Fig. 3Q).

Last, we aimed to comprehensively assess the convoluted shape of the Hindbrain Choroid Plexus by the implementation of the Landmark analysis into our pipeline. To achieve this, we first created the Hindbrain Choroid Plexus mean model of every analyzed developmental stage (Fig. 3R–T, green) from all available 3D Hindbrain Choroid Plexus models (Additional file 4: Fig. S2A-O). Then, we placed 10 landmarks across the whole 3D Hindbrain Choroid Plexus mean models as well as the individual 3D model to cover all parts of the Hindbrain Choroid Plexus tissue [e.g. ciliary zone, the rostral or caudal differentiated epithelium [2] (Fig. 3U)]. Analysis of the procrusion distance, which is the deviation of position between the same landmark placed on the 3D.

The Hindbrain Choroid Plexus mean model and the 3D model of evaluated individual Hindbrain Choroid Plexus displayed higher shape variability at E13.5 and E15.5 than the E17.5 (Fig. 3V).

### Detection of the morphological alterations in Hindbrain Choroid Plexus

To demonstrate the potential of Ch^O^P-CT, we applied the pipeline to our previously published µCT scans of the morphologically altered mutant embryos, namely the *Cdk13* hypomorphic (marked here as *Cdk13* HM) and *Cdk13* full knockout mutants (termed here as *Cdk13* KO) [24]. Considering the severe disturbance of the spectrum of organs including the brain accompanied by the embryonic lethality of these embryos [24] and the broad presence of the *Cdk13* transcripts within the Hindbrain Choroid Plexus epithelium, its progenitor zone as well as stroma (Additional file 4: Fig. S3A), we sought to explore whether these embryos displayed further brain defects, to-date undescribed morphological disruption in the Hindbrain Choroid Plexus tissue.

The classical histological analysis of the Hindbrain Choroid Plexus tissue marked by the epithelial marker *Transthyretin (Ttr)* [25] (Fig. 4A, in situ vertical panel) as well as the individual µCT scans (Fig. 4A, µCT slice vertical panel) and 3D model (Figs. 4A, 3D model vertical panel) of E13.5 WT, *Cdk13* HM as well as *Cdk13* KO are pointing to the gradual Hindbrain Choroid Plexus growth retardation within the mutant embryos. Indeed, the analysis of the 3 individual E13.5 Hindbrain Choroid Plexus of *Cdk13* HMs, as well as *Cdk13* KOs embryos (Additional file 4: Fig. S3B-G), uncovered a gradual decrease in the total volume (Fig. 4B) and area (Fig. 4C) of the Hindbrain Choroid Plexus in *Cdk13* HM, as well as *Cdk13* KO, compared to the WT Hindbrain Choroid Plexus. Conversely, we observed the increase of the 4th ventricle volume within the *Cdk13* KO embryos (Fig. 4D). Nonetheless, this shift still did not sufficiently impact the Hindbrain Choroid Plexus/ventricle volume ratio, as we detected the decreasing trend in the Hindbrain Choroid Plexus proportion in the total ventricle volume, which reached the statistical maximum in *Cdk13* KOs (Fig. 4E). The detailed volume assessment focused specifically on the two specific Hindbrain Choroid Plexus parts spreading along the rostrocaudal axis revealed the disruption of rostrocaudal symmetry of the developing Hindbrain Choroid Plexus within *Cdk13* KO (Fig. 4F). Further tendencies or statistically significant changes of the Hindbrain Choroid Plexus morphological disturbances within the *Cdk13* HM and *Cdk13* KO´s, respectively, encompasses the increase of the total outgrowth angle (Fig. 4G) and decrease in the total length of the Hindbrain Choroid Plexus branches (Fig. 4I), while the left–right symmetry is still preserved in these morphological parameters (Fig. 4H, J, respectively). The detailed length analysis of the individual Hindbrain Choroid Plexus branch parts determined the disruption in the extension of the Hindbrain Choroid Plexus lateral segments in the *Cdk13* KOs compared to the WTs (Fig. 4K).

Lastly, the Landmark analysis between the analyzed samples revealed the higher shape variability of *Cdk13* KOs samples, while the Hindbrain Choroid Plexus of *Cdk13* HMs was more similar to the corresponding mean model than the WT Hindbrain Choroid Plexus (Fig. 4L).

To show the ability of our tool to capture diverse morphological changes eventually caused by the disturbance of just one specific Hindbrain Choroid Plexus part or distinct cell type subpopulations, we further analysed the 3 Hindbrain Choroid Plexus of *Tmem107* full KO´s [26] (Additional file 4: Fig. S4A-C), as the transcripts of this gene can be found mainly in the ciliogenesis zone of the Hindbrain Choroid Plexus epithelium (Additional file 4: Fig. S4D). Neither the histological analysis of the Hindbrain Choroid Plexus tissue marked by the epithelial marker *Ttr* [25] (Fig. 5A, in situ vertical panel) nor the individual µCT scans (Fig. 5A, µCT slice vertical panel) accompanied by 3D models (Fig. 3D, 5A model vertical panel) of E15.5 *Tmem107* KO compared to WTs littermates did not uncover the striking morphological alternation at the first sight. Truly, the statistical analysis of all morphological parameters from our quantitative criterion panel (Fig. 5B–N) revealed the alternation just in the volume of the 4th ventricle of the *Tmem107* KO embryos (Fig. 5D) and outgrowth angle of the left Hindbrain Choroid Plexus branch (Fig. 5H), while the remaining parameters follow more or less the trends of the Hindbrain Choroid Plexus of E15.5 WTs (Fig. 5B, C, E–L). Nonetheless, considering the positive trend of the landmark analysis of the Hindbrain Choroid Plexus of *Tmem107* (Fig. 5L), we decided to modify this analysis by the selections of the landmarks placed in the regions with the most abundant presence of *Tmem107* transcripts (Fig. 5M). Positively, this adjustment revealed the shape alternations of the selected regions of the *Tmem107* KO Hindbrain Choroid Plexus compared to the WT littermates (Fig. 5N).

## Discussion

Organ spatiotemporal visualization and robust morphometrics are essential for a better understanding of the complex processes that occur during normal or pathological embryonic development. Here, we presented Ch^O^P-CT—the semiautomated platform that is primarily designed to segment the most shape-challenging form of the cerebrospinal fluid-secreting tissue—Hindbrain Choroid Plexus. Our MATLAB-based tool represents the simple, inexpensive and powerful method for the 3D visualization and subsequent morphology analysis of this convoluted organ using µCT scans. Thanks to the characteristics and the acquisition process of these types of data, our approach outstands among others, to-date used Choroid Plexus visualization as well as evaluation techniques, like whole-mount imaging [10] or the classical section immunostaining [6], which, in addition, do not usually provide the information about the whole Choroid Plexus tissue. Further elaborated, the Ch^O^P-CT-based Hindbrain Choroid Plexus 3D modelling and its further morphometrics are not burdened by the high autofluorescence, low antibody specificity or its penetration, fluorescence quenching or the necessity of the reporter gene usage or the pre-cleaning solutions. Also, this tool is not loaded by the time-demanding microscopy scanning of numerous tissue sections and further Hindbrain Choroid Plexus manual segmentation on several consecutive slices.

Ch^O^P-CT can be easily implemented. Firstly, it can efficiently work with any µCT-scans obtained with optimized scanning parameters, adequate resolution, and contrast, as were mentioned for our data. Nowadays, tremendous effort goes into the generation and phenotyping of thousands of knockout mice to unveil the function of every mouse gene. This effort under the umbrella of the International Mouse Phenotyping Consortium generates publicly available datasets and mouse strains that were already implemented in many studies by others [27, 28]. However, it seems that the µCT scans of mouse embryos available via The International Mouse Phenotyping Consortium webpage do not have sufficient resolution for the proper analysis of such small and complex tissue like the Hindbrain Choroid Plexus, as we indicated in this manuscript. Thus, we would like to emphasize the importance of high-resolution acquisition to allow the efficient data mining of these µCT datasets. On top of these requirements, the authors would like to mention the limits of the data type used in this manuscript. In detail, the µCT data do not provide insufficient resolution for the visualization of the Hindbrain Choroid Plexus microstructure, e.g. microvilli. Thus, the presented measurements do not capture the surface expansion caused by the formation of these structures on the apical side of the Hindbrain Choroid Plexus, as it was for instance described previously in the rat model [29]. Additionally, the sample preparation for the µCT measurement includes several steps, which lead to the shrinkage of the tissue [30, 31], thus the numbers presented in this study do not represent the absolute dimensions of this tissue and can not be directly compared to the values obtained from the diverse visualization methods. The authors would like to emphasize the importance of the exact timing during the sample preparation of the same developmental stage intended for the analysis of the Hindbrain Choroid Plexus outgrowth within the specific mutant embryos assessed by our tool.

Continuing the characterization of the Ch^O^P-CT tool, we would like to highlight the fact that future users can employ any type of open-source visualization software for the manual segmentation step or the morphometrics (e.g. 3D Slicer or Fiji). Bearing on this, we would like to recommend the users utilize the full criterion panel for the Hindbrain Choroid Plexus morphometric analysis presented here, to capture all nuances of this complex tissue. Moreover, we encourage the users to modify the given analysis (mainly the landmark analysis), according to the spatial profile of their protein of interest, as shown in the analysis of Hindbrain Choroid Plexus *Tmem107* KOs. Lastly, we would like to spotlight the possibility of downloading raw parameters and 3D models (details can be found in the section Material and Methods), which can significantly increase the user's initial analyzing groups and can potentially bring information about the Hindbrain Choroid Plexus biological variability among the various mouse strains used in the diverse research groups.

Using the Ch^O^P-CT pipeline and further manual measurements, we also pioneered the segmentation, 3D visualization and morphometric assessment of the whole embryonic Hindbrain Choroid Plexus of diverse WT embryonic stages. These analyses revealed the time-specific Hindbrain Choroid Plexus angular displacement and expansion of its lateral segments into the recesses of the 4th ventricle, pointing to the tightly regulated developmental time windows when Hindbrain Choroid Plexus possibly regulates the morphogenesis of the surrounding tissues. Noteworthy, the recent 3D reconstruction of the auditory system [32] highlighted the proximity of the Hindbrain Choroid Plexus to the auditory stem as a potentially novel regulatory factor in the formation of this body part. Thus, we recognise a comprehensive Hindbrain Choroid Plexus spatiotemporal characterization of the selected mutant embryos not only as the fundamental part in the identification of the Hindbrain Choroid Plexus impairment within these embryos but also in the detection of other potentially diminished brain regions.

On top of this, we also disclosed to date the undescribed central role of the *Cdk13* in the formation of the Hindbrain Choroid Plexus from the earliest stages of its development, which can significantly enhance the general knowledge about this understudied protein. Nonetheless, the mechanisms by which this kinase regulates the Hindbrain Choroid Plexus development remain unknown and need to be further properly investigated by the employment of several cell type-specific drivers in the researcher´s investigation, as the *Cdk13* is expressed globally within the Hindbrain Choroid Plexus tissue. Interestingly, the detailed analysis of the further mutant datasets of another poorly described gene—*Tmem107*—revealed almost no statistically significant changes in this tissue compared to the WT littermates, except mainly for the shape changes in the one specific Hindbrain Choroid Plexus zone, where this gene is mostly expressed. Moreover, this Hindbrain Choroid Plexus part termed a ciliogenesis zone, has been defined just recently by single-cell RNA sequencing [2] and the outcomes of its potential disruption on the Hindbrain Choroid Plexus functioning need to be further explored.

Ultimately, all the above-mentioned findings were obtained using the Ch^O^P-CT script as the core of the Hindbrain Choroid Plexus 3D visualization and its morphometric examination supplemented by the manual tuning of these steps. Thus, our tool has been proven to be beneficial not only for basic Hindbrain Choroid Plexus biology but also indicated enormous potential in the unbiased identification of the genes involved in the Hindbrain Choroid Plexus and consequently other surrounding neural tissue development.

## Conclusions

The publicly accessible Ch^O^P-CT tool introduced in this manuscript represents an effective instrument for the Hindbrain Choroid Plexus 3D visualization and its quantitative morphology assessment in mouse embryos. Thus, Ch^O^P-CT has the potential to standardize and significantly boost the research on Choroid Plexus and facilitate the unbiased identification of the key genes, which guide the proper Hindbrain Choroid Plexus development.

## Supplementary Information

**Additional file 1.** 3D PDF - E13.5

**Additional file 2.** 3D PDF - E15.5

**Additional file 3.** 3D PDF - E17.5

**Additional file 4:** Supplementary Materials.

## Data Availability

All measured parameters, as well as the.stl files of the mean models, have been deposited in the National Repository (https://data.narodni-repozitar.cz) under the 10.48700/datst.z0d7f-xt218. The Ch^O^P-CT script is available on the Github webpage of the Ceitec CT laboratory (https://github.com/CEITEC-CTLAB) in the repository called Ch^O^P-CT. Videotutorial can be found on the Youtube platform under the name "Ch^O^P-CT".

## Abbreviations

µCT: X-ray micro-computed tomography
3D: Three dimensional
Ch^O^P-CT: Ch^O^Pping the Ch^O^roid Plexus out of the µCT data
E: Embryonic day
HM: Hypomorphic mutant
KO: Knockout
WT: Wild-type
