# Supplementary material for: ChOP-CT: quantitative morphometrical analysis of the Hindbrain Choroid Plexus by X-ray micro-computed tomography
Source: Fluids Barriers CNS. 2024 Jan 24;21:9. doi: 10.1186/s12987-023-00502-8 (PMC11406807; doi:10.1186/s12987-023-00502-8)
Supplement: Supplementary file 2 — Additional file 2. 3D PDF - E15.5 [file 12987_2023_502_MOESM2_ESM.pdf]

Interactive 3D reconstruction of a Hindbrain Choroid Plexus (HbChP) – E15.5

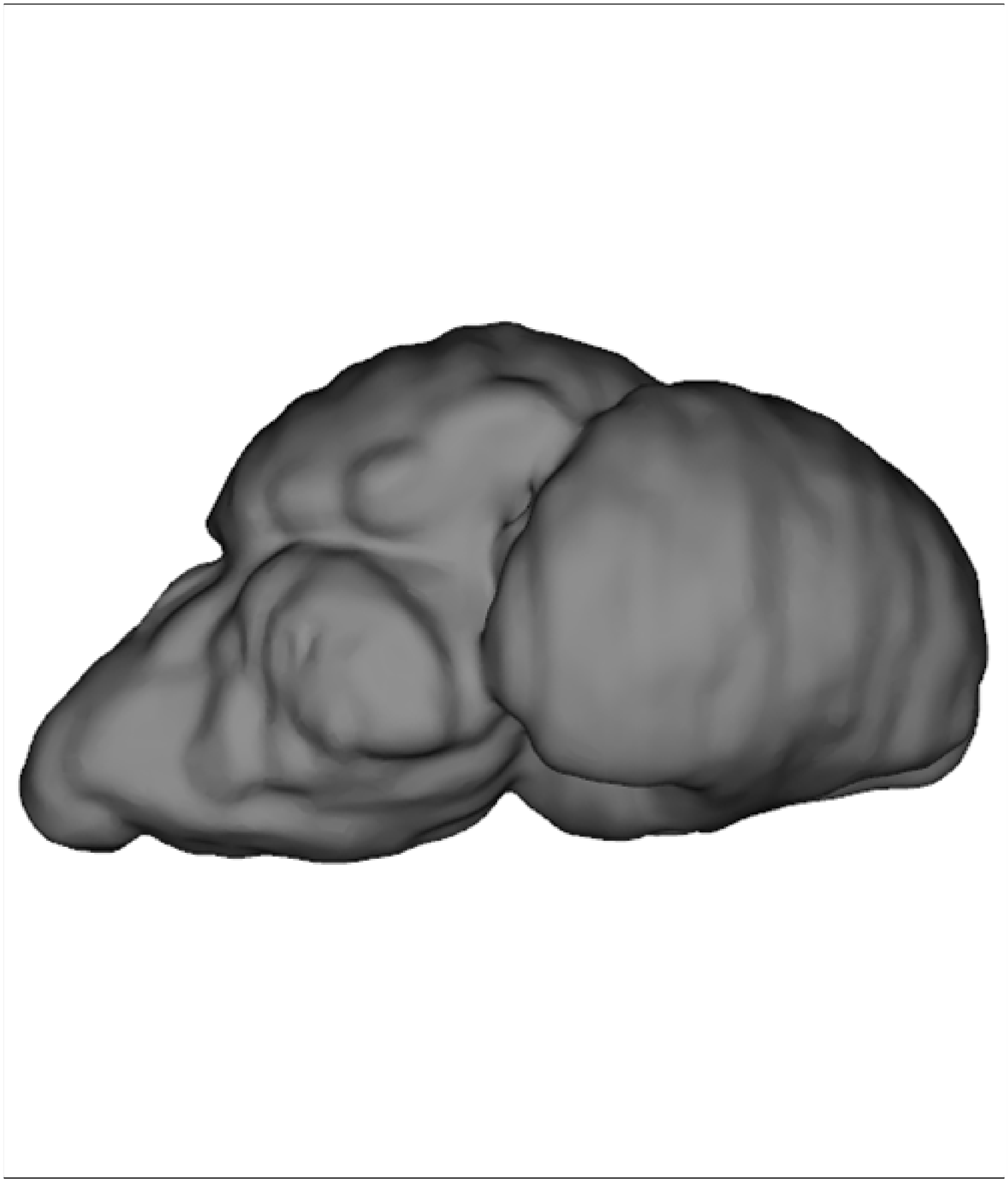

HbChP, Brain, Vertebrate brain ventricles

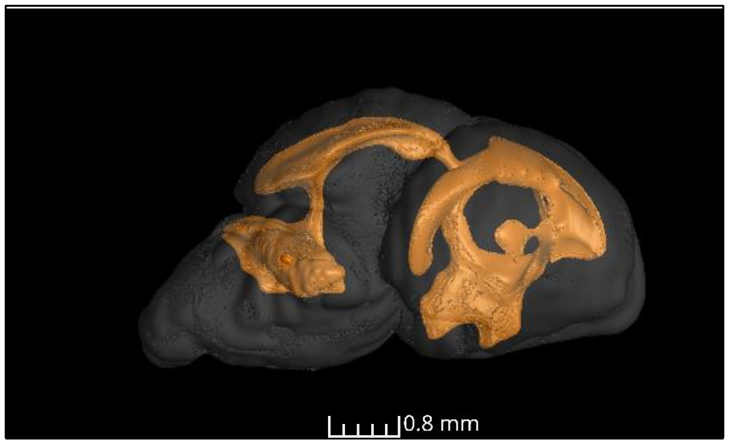

HbChP, Vertebrate brain ventricles

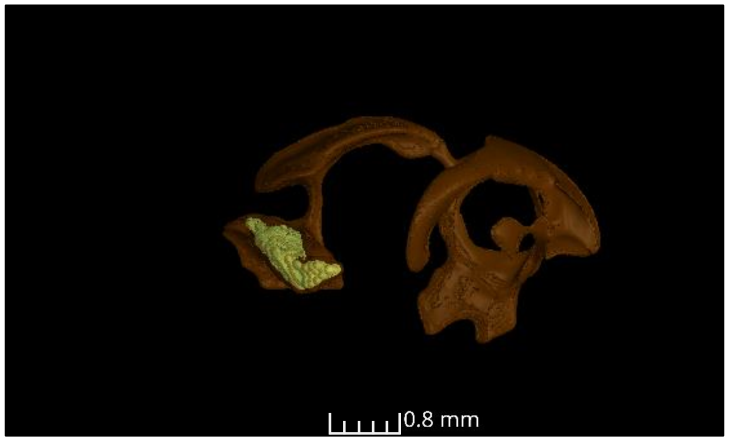

HbChP

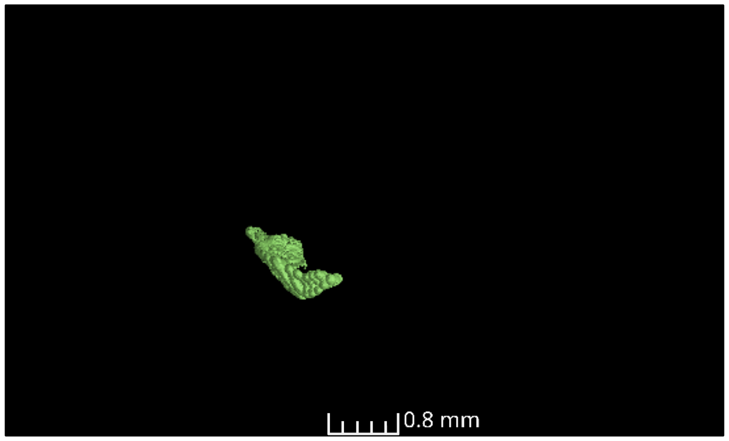

Technical note

To view interactive PDF files, you need to use the free standard Adobe Reader®/Acrobat Reader DC (www.adobe.com/downloads/). If you see a warning message in the yellow dialog box on the file opening, you can activate the content by pressing the button "Options", otherwise you can enable 3D data for all documents in the preferences dialog box (Edit / Preferences / 3D & Multimedia / check the box "Enable playing of 3D content").
