# Supplementary material for: ChOP-CT: quantitative morphometrical analysis of the Hindbrain Choroid Plexus by X-ray micro-computed tomography
Source: Fluids Barriers CNS. 2024 Jan 24;21:9. doi: 10.1186/s12987-023-00502-8 (PMC11406807; doi:10.1186/s12987-023-00502-8)
Supplement: Supplementary file 4 — Additional file 4: Supplementary Materials. [file 12987_2023_502_MOESM4_ESM.pdf]

Supplementary Materials for

ChOP-CT - Quantitative morphometrical analysis of the Hindbrain Choroid Plexus by X-ray micro-computed tomography.

Viktória Parobková & Petra Kompaníková *et al.*

*Corresponding authors. emails: tomas.zikmund@ceitec.vutbr.cz, bryja@sci.muni.cz

This PDF file includes:

Supp. Figs. 1 to 4
Supp. Tables 1 to 3

Other Supplementary Materials for this manuscript include the following:

Interactive pdfs Additional.pdf_A to Additional.pdf_C

Supp. Fig.1

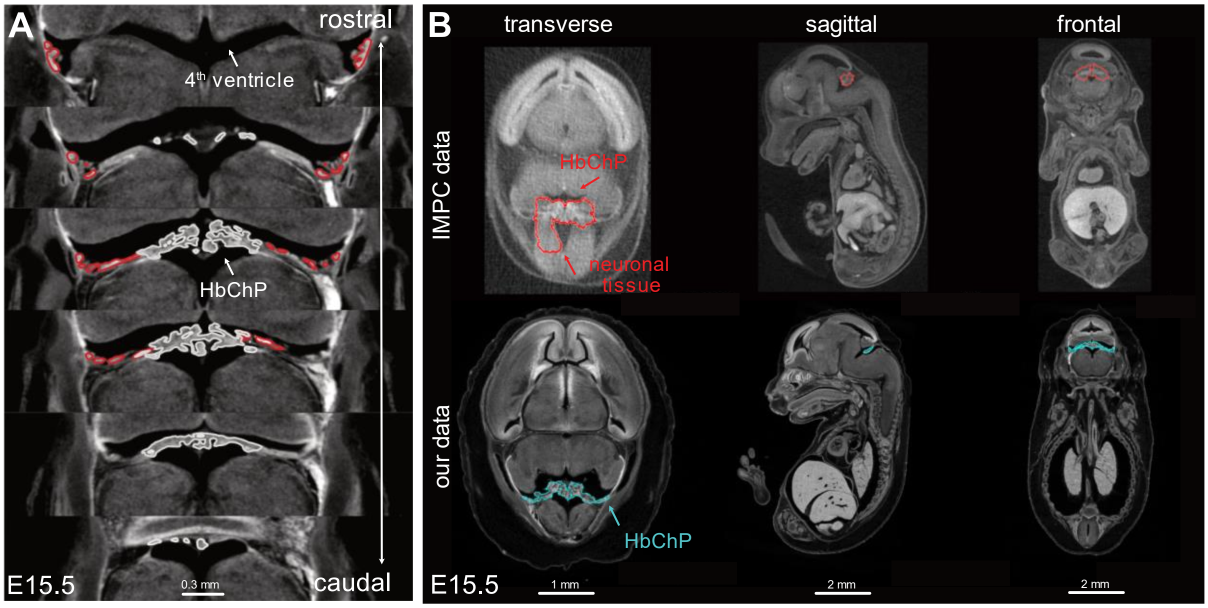

Supp. Fig. 1.: The effectiveness of the Ch^{O}P-CT tool on various data

(A) High power frontal view on sections of E15.5 HbChP from Lugol-stained embryo, which was visualized by μCT. HbChP parts segmented by the Ch^{O}P-CT tool are highlighted in grey colour. The HbChP parts, which must be segmented manually, are visualized in red. Scale bar: 0.3 mm. **(B)** The comparison of the μCT data from IMPC (top horizontal panel) and our laboratory (bottom horizontal panel) presenting the significant inaccuracies in the Ch^{O}P-CT-based HbChP segmentation caused by the low-resolution data from IMPC (red colour) compared to segmentation on our dataset (blue colour). Both datasets are presented by one high-power transverse (left vertical panel), sagittal (middle vertical panel) and frontal (right vertical panel) μCT section of E15.5 Lugol-stained embryo visualized μCT. Scale bar: transverse views: 1 mm, sagittal and frontal views: 2 mm. Abbrevations: μCT - X-ray micro-computed tomography, E- embryonic day, HbChP - Hindbrain Choroid Plexus, Ch^{O}P-CT - Ch^{O}Pping the Ch^{O}roid Plexus out of the μCT data, IMPC - International Mouse Phenotyping Consortium, WT – wild-type.

Supp.Fig.2

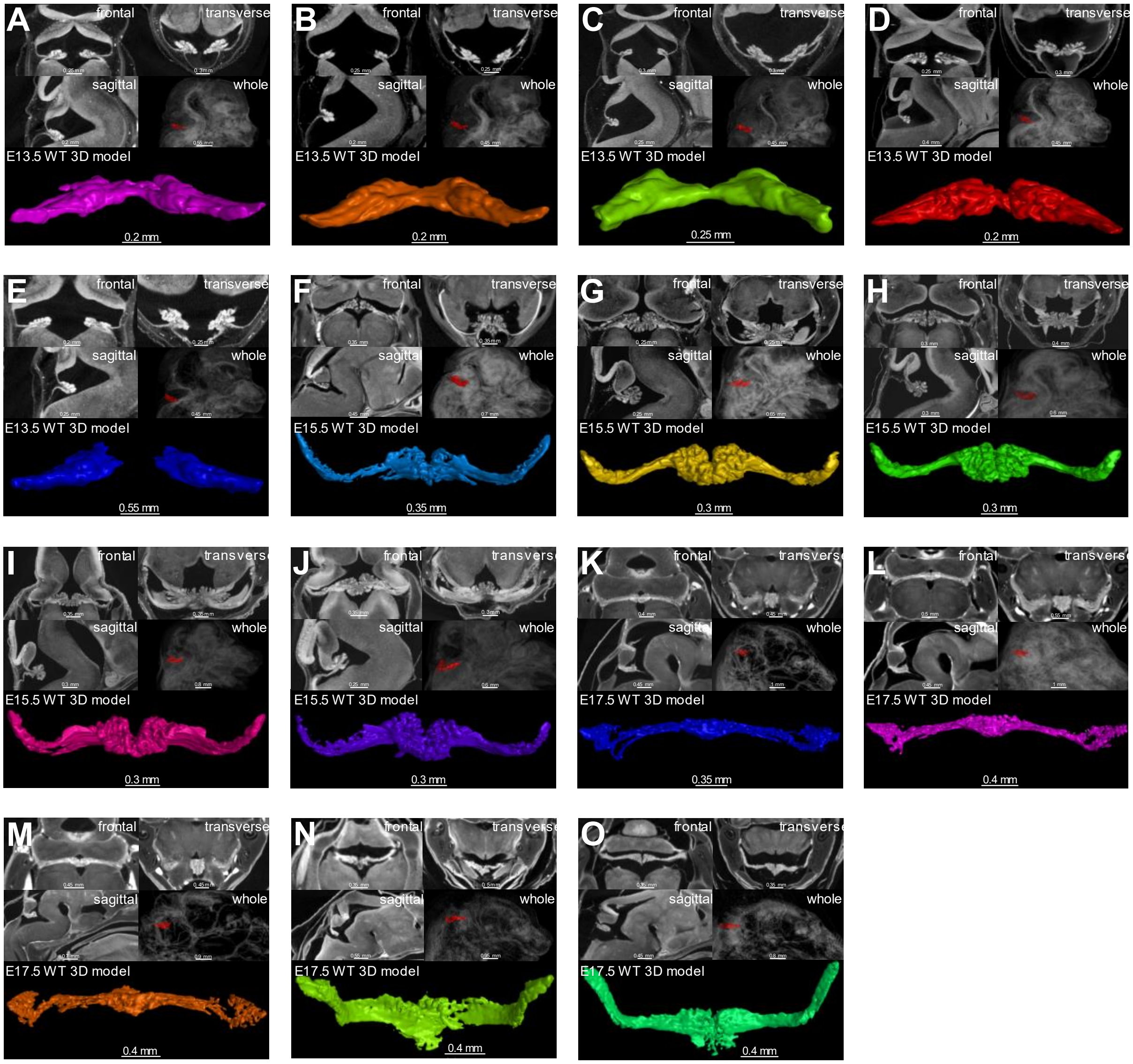

Supp. Fig. 2.: Visualization of all WT samples used in the study
(A-I) All WT Lugol-stained embryos visualized by µCT and corresponding 3D model of HbChP tissues used for the morphological analysis. Every biological replicate of every analyzed developmental stage (E13.5: A-E, E15.5: F-J, E17.5: K-O) is always visualized by high-power frontal, transverse and sagittal section of the dorsal head regions with the HbChPs. Additionally, the 3D model of the HbChP is presented not only separately but also within the dorsal part of the sagittally placed embryo´s head (global view) giving the HbChP spatial information within the developing embryo. Scale bars can be found within the individual images. Abbrevations: 3D – three dimensional, E- embryonic day, HbChP - Hindbrain Choroid Plexus, µCT - X-ray micro-computed tomography, WT – wild-type.

Supp.Fig.3

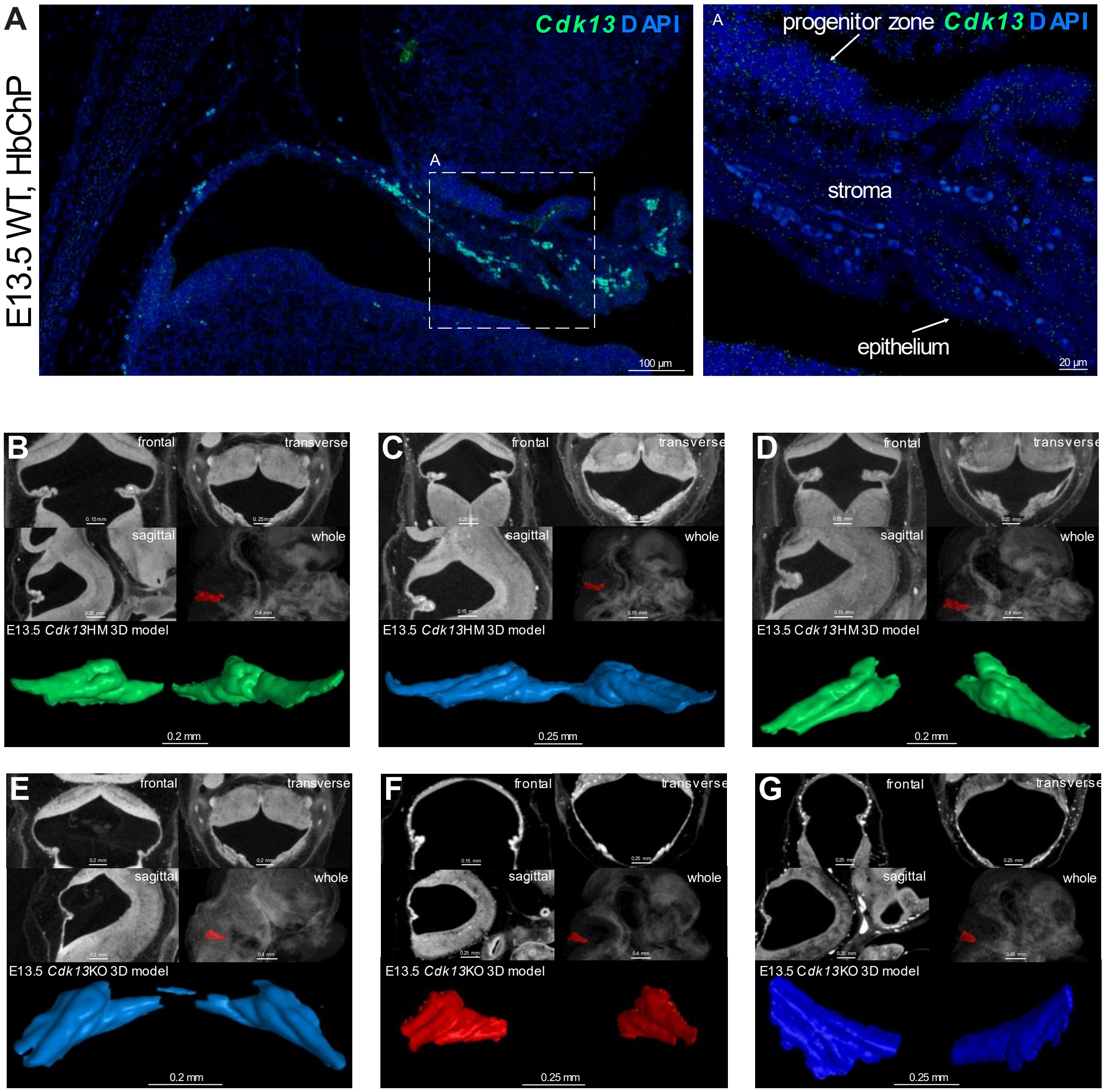

Supp. Fig. 3.: Visualization of *Cdk13* within the HbChP and all *Cdk13* HM and KO samples used in the study

(A) *In situ* analysis of the *Cdk13* transcripts (green dots) within the E13.5 WT HbChP. The close-up (A) is showing the presence of *Cdk13* within the HbChP epithelial progenitor zone, epithelium as well as its stroma. *In situ* analysis was performed on the frontal sections. Scale bars: main figure: 100 µm, close-up: 20 µm. **(B-G)** All E13.5 *Cdk13* HMs (B-D) and *Cdk13* KOs (E-G) Lugol-stained embryos visualized by µCT and corresponding 3D model of HbChP tissues used for the morphological analysis. Every biological replicate is always visualized by the high-power frontal, transverse and sagittal section of the dorsal head regions with the HbChPs. Additionally, the 3D model of the HbChP is presented not only separately but also within the dorsal part of the sagittally placed embryo´s head (global view) giving the HbChP spatial information within the developing embryo. Scale bars can be found within the individual images. Abbrevations: 3D – three dimensional, E- embryonic day, HbChP - Hindbrain Choroid Plexus, HM - hypomorphic mutant, µCT - X-ray micro-computed tomography, KO - knock-out, WT - wild-type.

Supp.Fig.4

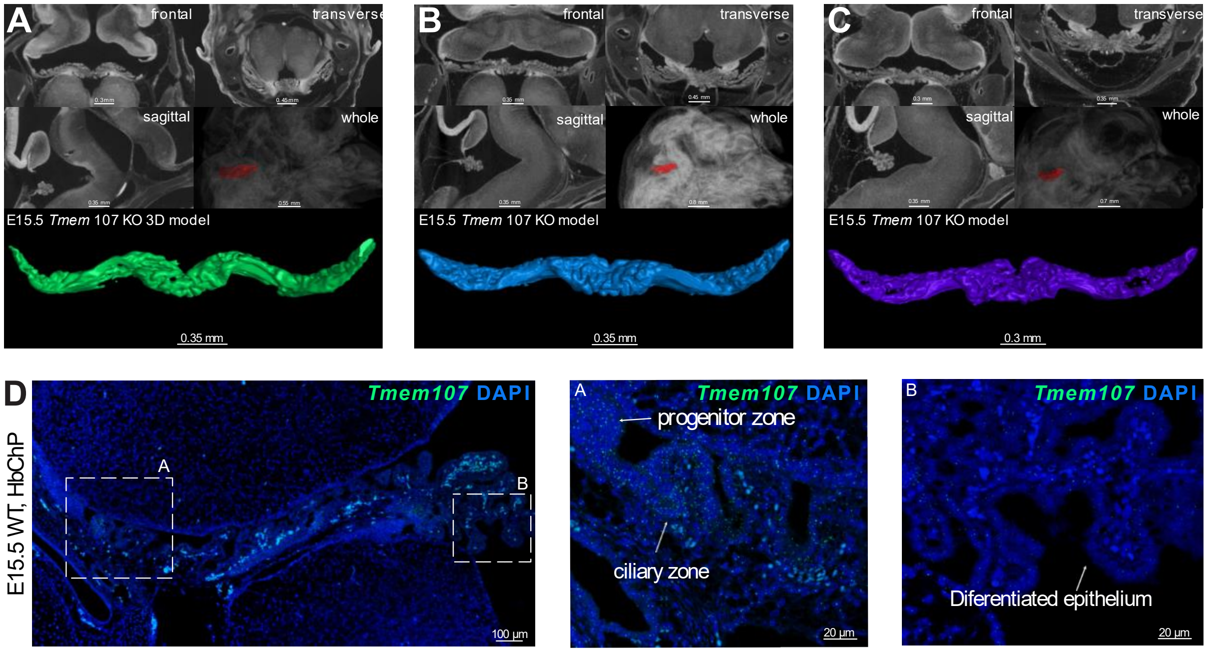

Supp. Fig. 4.: Visualization of *Tmem107* within the HbChP and all *Tmem107* samples used in the study

(A-C) All E15.5 *Tmem107* KOs Lugol-stained embryos visualized by µCT and corresponding 3D model of HbChP tissues used for the morphological analysis. Every biological replicate is always visualized by the high-power frontal, transverse and sagittal section of the dorsal head regions with the HbChPs. Additionally, the 3D model of the HbChP is presented not only separately but also within the dorsal part of the sagittally placed embryo´s head (global view) giving the HbChP spatial information within the developing embryo. Scale bars can be found within the individual images. **(D)** *In situ* analysis of *Tmem107* transcripts (green dots) determined its presence within the E15.5 WT HbChP. Detailed view (A) displays the higher *Tmem107* abundance within the HbChP epithelial ciliary zone compared to the already differentiated HbChP epithelium within the central part of this tissue (detail B). Scale bar: main figure: 100 µm, close-ups: 20 µm. Abbrevations: 3D – three dimensional, E- embryonic day, HbChP - Hindbrain Choroid Plexus, µCT - X-ray micro-computed tomography, KO - knock-out , WT - wild-type.

Supp. Table 1.:The key resources, page 1

REAGENT or RESOURCE	SOURCE or DESCRIPTION	IDENTIFIER or VERSION
**Experimental Mouse models**		
Cdk13 HM	Novaková et al., 2019	NA
Cdk13 KO	Novaková et al., 2019	NA
Tmem107 KO	Christopher et al., 2012	NA
**Chemicals, kits, probes and fluorescence dyes**		
Agarose	Top-BIO	P045
DAPI	ACD Bio	323108
Ethanol (EtOH)	Penta	71250-11001
FluoroshieldTM	Sigma-Aldrich	F6059
Hydrogen peroxide (H_2O_2)	Merck	SIAL95294
I_2	Penta	17570-30250
Methanol (MeOH)	Penta	21210-11000
Paraffine	Penta	HI-00403
Paraformaldehyde (PFA) (4%)	Sigma	P6148
Protease	ACD Bio	322331

Supp. Table 1.: The key resources, page 2

RNAscope® Multiplex Fluorescent v2 Assay kit	ACD Bio	323110
RNAscope® Target Retrieval Reagent	ACD Bio	322000
TSA Plus Cyanine 3	Perkin Elmer	NEL744001KT
Xylene	Penta	601-022-009
**Machines, software & algorithms**		
Avizo	Thermo Fisher Scientific	9.5
Fluorescence microscope	Zen	LSM800
GE Phoenix datos|x	Waygate Technologies GmbH	2.0
GE Phoenix v|tome|x	Waygate Technologies, Baker Hughes Digital Solutions GmbH	L 240
Generalized-Procrustes-analysis	Pulak Purkait	N/A
GraphPad Prism	Dotmatics	9.0.0
Matlab	MathWorks	2022b
VGStudio MAX	Volume Graphics GmbH	4.1
Zen Blue	Zen	3.5

Supp. Table 2.: Preparation of fixed embryos for µCT scanning

Developmental stage	Incubation time (h)								
	Dehydration						Staining	Washing	
	10% EtOH	30% EtOH	50% EtOH	70% EtOH	80% EtOH	90% EtOH	1% I_2	30% EtOH	10% EtOH
E13.5	1	1	1	1	1	1	8	1	1
E15.5	4	4	4	4	4	4	18	1	1
E17.5	8	8	8	8	8	8	72	1	1

Supp. Table 3.: The scanned voxel size of the measured samples

E13.5 embryos	Voxel size (mm)	E15.5 embryos (mm)	Voxel size	E17.5 embryos (mm)	Voxel size
WT #1	0,0043	WT #1	0,0055	WT #1	
WT #2	0,0043	WT #2	0,0045	WT #2	
WT #3	0,0044	WT #3	0,0045	WT #3	
WT #4	0,0044	WT #4	0,0032	WT #4	
WT #5	0,0044	WT #5	0,0036	WT #5	
Cdk13 HM #1	0,0043	*Tmem107* KO #1	0,0045		
Cdk13 HM #2	0,0044	*Tmem107* KO #2	0,0036		
Cdk13 HM #3	0,0044	*Tmem107* KO #3	0,0036		
Cdk13 KO #1	0,0043				
Cdk13 KO #2	0,0044				
Cdk13 KO #3	0,0044				
